# Sperm DNA fragmentation testing in clinical management of reproductive medicine

**DOI:** 10.1002/rmb2.12547

**Published:** 2023-10-31

**Authors:** Ava Adler, Bradley Roth, Scott D. Lundy, Teppei Takeshima, Yasushi Yumura, Shinnosuke Kuroda

**Affiliations:** ^1^ Glickman Urological & Kidney Institute Cleveland Clinic Foundation Cleveland Ohio USA; ^2^ Department of Urology, Reproduction Center Yokohama City University Medical Center Yokohama Japan

**Keywords:** clinical practice, DNA repair, Infertility, reproductive outcomes, sperm DNA fragmentation

## Abstract

**Background:**

Approximately 8%–12% of couples worldwide face infertility, with infertility of individuals assigned male at birth (AMAB) contributing to at least 50% of cases. Conventional semen analysis commonly used to detect sperm abnormalities is insufficient, as 30% of AMAB patients experiencing infertility show normal results in this test. From a genetic perspective, the assessment of sperm DNA fragmentation (SDF) is important as a parameter of sperm quality.

**Methods:**

In this narrative study, we review and discuss pathophysiological causes, DNA repair mechanisms, and management of high SDF. We then summarize literature exploring the association between SDF and reproductive outcomes.

**Main Findings:**

Recent systematic reviews and meta‐analyses have revealed a significant association between high SDF in AMAB individuals and adverse reproductive outcomes including embryo development, natural conception, intrauterine insemination, and in vitro fertilization. However, the association with live birth rates and pregnancy rates following intracytoplasmic injection remains inconclusive. The disparities among quantitative assays, inconsistent reference range values, absent high‐quality prospective clinical trials, and clinical heterogeneity in AMAB patients with elevated SDF represent the main limitations affecting SDF testing.

**Conclusion:**

The evaluation and management of SDF plays an important role in a subset of AMAB infertility, but widespread integration into clinical guidelines will require future high‐quality clinical trials and assay standardization.

## Graphical abstract

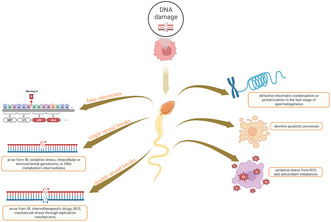

## 1 INTRODUCTION

Infertility presents as a complex diagnosis affecting all patients seeking biological children. Globally, approximately 8%–12% of couples experience infertility, which the WHO defines as the inability to conceive after a year of consistent unprotected intercourse.^1^ Research consistently highlights that roughly 50% of these failures to conceive are associated with sperm‐related factors.^2^ In humans, successful fertilization depends on the integration of paternal genomes from haploid sperm cells with maternal DNA from the oocyte, ensuring transmission of a properly functioning genome to the next generation. The efficient production of healthy mature spermatozoa through spermatogenesis and spermiogenesis is a continuous and essential process for the fertility of individuals assigned male at birth (AMAB).^3^

A comprehensive andrological consultation and examination are essential for all AMAB individuals seeking assistance for infertility. The standard evaluation for infertility typically involves a semen analysis (SA), which assesses sperm production, motility, and viability. However, SA lacks the ability to determine the functional potential of sperm to fertilize an oocyte and contribute to embryonic development.^4^ While SA can determine abnormal semen conditions like asthenozoospermia, oligozoospermia, and azoospermia, this test cannot definitively determine fertility or infertility in all patients.^5^, ^6^ The WHO has established reference values for SA parameters arbitrarily based on 5th percentile cutoffs from a large population from various countries. However, the predictive value of these reference values has been a subject of ongoing debate, as no single or set of semen parameters is highly indicative of AMAB fertility.^7^, ^8^ Moreover, SA exhibits considerable variability over time, underscoring the need for at least two SA tests to ensure a thorough evaluation.^4^ Moreover, relying solely on reference semen analysis values to support natural conception is no longer applicable in the era of in vitro fertilization (IVF)/intracytoplasmic injection (ICSI). These assisted reproductive technologies (ART) require fewer but arguably higher‐quality spermatozoa for successful pregnancies and live births.^8^ Idiopathic infertility is widely recognized as the primary cause of AMAB infertility, accounting for approximately 40%–50% of cases.^2^ However, since up to 30% of infertile patients AMAB exhibit normal results in conventional SA, this method does not capture all relevant information and cannot be solely relied upon to detect sperm abnormalities.^9^, ^10^ Consequently, there is a growing demand for objective parameters and seminal markers to improve infertility diagnosis. Some laboratories have incorporated advanced sperm tests, such as assessing acrosome functionality, chromatin condensation, or DNA fragmentation, into their andrological evaluations.

Sperm DNA fragmentation (SDF) is a parameter related to AMAB fertility that conventional SA fails to directly identify. The rationale behind SDF testing is to assess the genetic integrity of spermatozoa. SDF can occur due to defective chromatin condensation or protamination during spermiogenesis, unsuccessful apoptotic processes, and oxidative stress caused by imbalances in reactive oxygen species (ROS) and antioxidants.^11^, ^12^ Numerous studies have demonstrated that infertile AMAB patients often have elevated SDF levels associated with various risk factors including smoking, drug use, and lifestyle choices.^13^, ^14^ Recently, the WHO laboratory manual included SDF as an extended examination and provided detailed procedures for its assessment.^4^ Global guidelines now increasingly recommend measuring SDF in specific situations including recurrent pregnancy loss (RPL) and cases of “unexplained” AMAB infertility, especially when conventional SA yields normal results.^15^, ^16^

Despite this recommendation, the results of DNA fragmentation studies are not consistently reliable due to various factors. These factors include the absence of standardized cutoff values to differentiate between fertile and infertile patients or to predict reproductive outcomes, non‐standardized protocols measuring a mix of single and double strand breakages, the use of different testing assays that measure unrelated parameters for assessing DNA fragmentation, and the lack of randomized controlled trials (RCTs) assessing efficacy.^17^ Thus, the question of whether SDF measurement should be routinely incorporated into fertility evaluations remains a topic of debate. Nevertheless, SDF prevails as a potential objective biomarker for diagnosing and evaluating AMAB infertility and guiding treatment decisions.^18^ The purpose of this narrative review is to provide an updated summary of the current evidence concerning SDF measurement in clinical practice and its impact on clinical decision‐making. In addition, we aim to explore the pathophysiological aspects of SDF, including its underlying causes, repair mechanisms, and strategies for managing high SDF.

## 2 MATERIALS AND METHODS

This narrative review utilized the PubMed/MEDLINE database for conducting the literature search. The search strategy encompassed several key terms, such as “sperm DNA fragmentation/damage,” “DNA fragmentation index,” “male infertility,” “spontaneous pregnancy,” “reproductive outcome,” “recurrent pregnancy loss,” “sperm DNA repair,” and “artificial reproductive technologies.” The comprehensive literature search was conducted from February 1st 2023 to April 30th 2023. Articles were included in this review if they were written in the English language and if their titles or abstracts were relevant to the context of this review. Comments, editorials, and non‐English articles were excluded from consideration. Additionally, relevant articles were manually identified from the reference lists of the included articles.

## 3 MAIN FINDINGS

### 3.1 Molecular mechanisms of SDF

**FIGURE 1:**
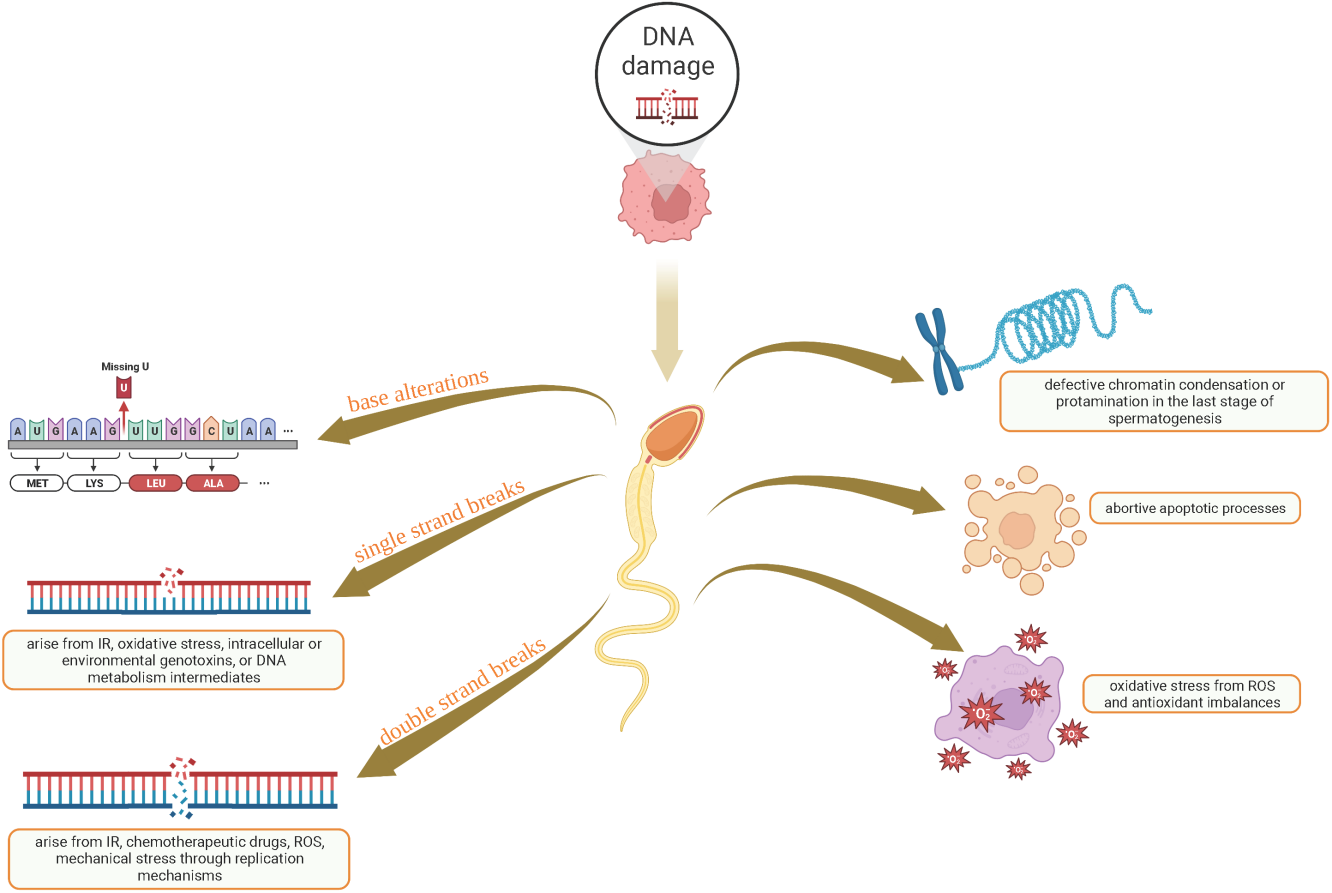
Summary of various types and underlying molecular mechanisms of sperm DNA damage.

The presence of sperm DNA damage in the ejaculate can be attributed to three different mechanisms: defective chromatin condensation or protamination during the last stage of spermatogenesis, abortive apoptotic processes, and oxidative stress resulting from imbalances in ROS and antioxidants.^11^ DNA damage manifests as structural changes including base alterations, single‐strand breaks (SSBs), and double‐strand breaks (DSBs) and are summarized in Figure 1.

SSBs are the most prevalent DNA lesions and can arise from various sources such as ionizing radiation (IR), oxidative stress, intracellular or environmental genotoxins, or intermediates of normal DNA metabolic processes. These breaks are detected by poly (ADP‐ribose) polymerase 1 (PARP1) and 2 (PARP2), leading to enzymatic activation and the synthesis of poly (ADP‐ribose) (PAR) chains. This activation triggers the mono‐ and poly‐ADP‐ribosylation of target proteins and surrounding histones, which are subsequently recycled for successive SSB mechanisms. The activation of PARP following SSB detection plays a crucial role in regulating chromatin structure and compaction, transcription, and the recruitment of proteins involved in SSB repair.^18^, ^19^, ^20^ Following SSB detection by PARP1/2, various end‐processing proteins including XRCC1 protein complexes such as PNKP, APE1, and APE2 are recruited through interaction with XRCC1 and poly (ADP‐ribose). This recruitment leads to the restoration of 3′‐hydroxyl (OH) termini breaks through PKNP phosphatase and kinase activity, converting 3′‐P and 5′‐OH termini breaks into 3′‐OH and 5′‐P termini. This allows for DNA repair synthesis by DNA polymerases and DNA‐end joining by DNA ligases.^21^

DSBs are severe DNA lesions that can arise from either exogenous sources such as chemotherapeutic drugs or endogenous sources (e.g. ROS, mechanical stress, and replication aberrations). In specific cellular processes like meiosis, intentional DSBs are generated by nucleases to initiate homologous chromosome recombination. DSBs are highly dangerous and can trigger apoptosis since their repair mechanisms are more challenging than other types of DNA damage; they cannot utilize a sister strand as a template for repair.

Rejoining the broken strands through ligation can result in carcinogenic mutations and translocations or even lead to tumorigenesis. Notably, several lymphatic‐originating cancers exhibit chromosomal rearrangements involving immunoglobulin or T‐cell receptor loci, suggesting that these cancers may have originated from inadequate resolution of DSBs initiated during V(D)J recombination.^22^, ^23^, ^24^

The discovery of H2AX (gH2AX) as a potential biomarker for cellular responses to DSB has been crucial in understanding DNA damage response (DDR) mechanisms. When DSBs occur, phosphorylation of the Ser‐139 residue of the histone variant H2AX by members of the phosphatidyl‐inositol‐3‐kinase‐related kinases (PIKKs) family takes place, resulting in the formation of gH2AX. This modification enhances DNA accessibility through chromatin modification, which leads to the amplification of proteins involved in the DDR signaling cascade.^20^, ^24^, ^25^ Cells lacking H2AX have been observed to exhibit slower repair processes and more chromosomal rearrangements than cells with H2AX present, highlighting the significant role played by gH2AX in maintaining genomic stability. However, despite its importance, the lack of standardized parameters and technical heterogeneity in quantifying gH2AX limits its utility as a biomarker for DNA damage or repair, and further research on gH2AX as a biomarker is necessary to establish its clinical application.^26^

Two complementary repair mechanisms have evolved to counteract the damaging effects of DSBs: homologous recombination (HR) and non‐homologous end‐joining (NHEJ). The choice between these repair pathways depends on the cell cycle stage and the extent of DNA breakage. HR primarily operates during DNA replication and post‐replication in the S and G2 phases of the cell cycle when non‐damaged repair templates are available. This process is considered error‐free and preserves genomic information. The repair is initiated by the MRE11‐RAD50‐NBS1 (MRN) complex, which detects and binds to the DSB to bring the ends together. The serine/threonine kinase ATM (ataxia telangiectasia mutated) is recruited upon complex binding and phosphorylates key proteins, including γ‐H2AX, which plays roles in DNA damage checkpoints and catalyzing repair. The 5′‐ends of the DSBs are then nucleolytically cleaved, creating 3′ overhangs. RAD51 generates a nucleoprotein that forms a D‐loop conformation, connecting and synthesizing DNA strands.^23^, ^24^, ^27^ In contrast, NHEJ repairs DSBs by directly rejoining the two broken ends without using a homologous sequence as a template. During various cell cycle stages, NHEJ is the dominant repair mechanism, especially in G0 and G1 phases and during V(D)J recombination and immunoglobulin recombination in lymphocytes. In NHEJ repair, the two broken ends are rejoined through the action of the DNA‐end binding protein Ku, which recruits DNA‐dependent protein kinase catalytic subunits (DNA‐PKcs). DNA‐PKcs then recruit XRCC4 and ligase IV, facilitating the re‐ligation of the broken ends. This repair process is faster but less accurate than HR. NHEJ often leads to the loss of a few nucleotides at each broken end and may result in insertions and deletions, making it an error‐prone mechanism that does not precisely preserve genomic information due to the lack of a homologous template.^20^, ^22^, ^23^, ^24^, ^27^

### 3.2 Natural sperm DNA repair mechanisms

Spermatozoa are remarkably resilient cells, enduring the challenging journey from the epithelial testes to the fallopian tubes to reach the oocyte. Mammalian sperm DNA structure is highly compacted and unlike any other eukaryotic cell. This structure is achieved through specialized sperm chromatin packaging, which maintains DNA integrity and organization from spermatogenesis until fertilization. The compaction process occurs during spermiogenesis, wherein histones are partially replaced by protamines—proteins rich in arginine and cysteine. The DNA‐protamine complex structure involves protamines binding to the minor groove of DNA, neutralizing the backbone, forming hydrogen, hydrophobic, and disulfide bonds between each protamine at every DNA turn. This configuration effectively renders the chromatin invulnerable to external attacks, providing protection for the DNA.^28^, ^29^, ^30^, ^31^ However, despite this safeguarding mechanism, the DNA is still prone to damage in this compacted state.^32^, ^33^ Protamine deficiencies show an inverse correlation with DNA fragmentation,^34^ and insufficient nucleosome‐to‐protamine remodeling is associated with infertility.^35^

DNA replication is an integral process in all living organisms, yet DNA is highly vulnerable to damage. Damage can occur due to oxidative stress, spontaneous replication errors, and cellular metabolism, leading to the generation of as many as 10^5^ lesions per cell per day.^36^ Several theories regarding the causes of sperm DNA damage have been proposed and include ROS damage, sperm chromatin package defects, and abortive apoptosis. Multiple studies have now identified a positive correlation between ROS levels and DNA fragmentation.^37^ While maintaining a baseline level of physiological ROS appears to decrease DNA fragmentation and be essential for oocyte‐sperm fusion (capacitation), elevated ROS levels lead to higher rates of DNA fragmentation, showing an inverse correlation with semen volume, the percentage of motile spermatozoa, and sperm linearity.^38^, ^39^

**FIGURE 2:**
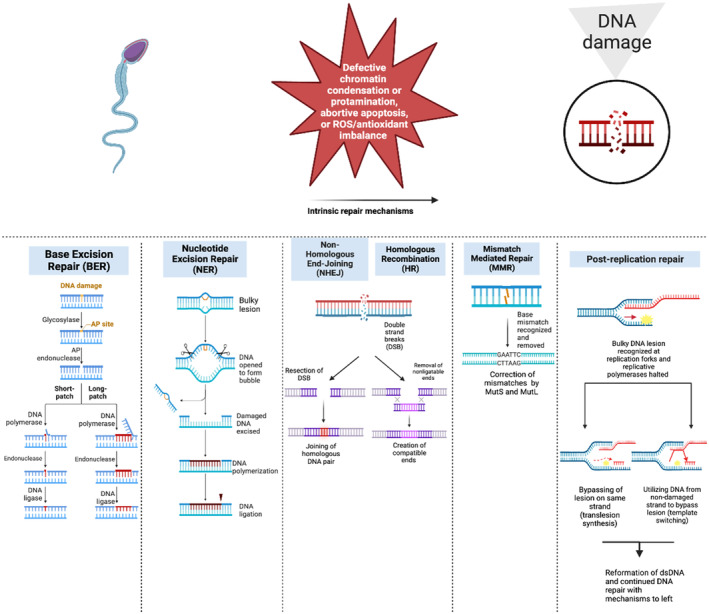
Summary of natural sperm DNA repair mechanisms.

To counteract DNA damage in spermatozoa, five natural repair mechanisms have evolved. These mechanisms are nucleotide excision repair (NER), base excision repair (BER), DNA mismatch repair (MMR), DSB repair mechanisms, and post‐replication repair (PRR). NER primarily removes bulky lesions, such as pyrimidine or thymine dimers caused by UV exposure, as well as bulky adducts and DNA intra‐strand cross links that distort the helix's structure. XPC/RAD23B proteins scan for damage detection in this process. On the contrary, BER is responsible for repairing non‐helix distorting lesions, particularly those resulting from deamination, oxidation, and free radical damage. The BER mechanism involves DNA glycosylases that recognize and remove damaged bases by cleaving the N‐glycosidic bond to deoxyribose. MMR functions as a proofreader during DNA replication, identifying base–base mismatches and insertion–deletion loops through antigen proteins MutS and MutL. This process significantly increases DNA replication stability by about 100‐fold. Among various types of DNA damage, DSBs are the most harmful and can result from factors like ROS, failed DNA replication, repair, and recombination, meiosis, as well as exposure to IR or chemotherapeutic agents. The repair pathways for DSBs include HR and NHEJ. HR primarily resolves DSBs during the S and G2 phases of the cell cycle, using the MRN complex comprising MRE11, RAD50, and Nijmegen breakage syndrome 1 (Nbs 1) proteins. NHEJ, on the contrary, creates compatible ends in DSBs through Ku heterodimers, which recruit several kinases and enzymes for the ligation process.^40^, ^41^, ^42^, ^43^, ^44^ PRR, working at the replication fork level, facilitates ongoing synthesis of damaged genomes. To prevent replication fork stalls, PRR employs trans‐lesion synthesis (TLS) and template switching (TS). TLS involves specialized polymerases that replicate directly across lesions, whereas TS operates by utilizing genetic information from the newly synthesized chromatid as a template for replication, effectively avoiding the potential for fork‐stalling lesions.^19^ Natural sperm DNA repair mechanisms are summarized in Figure 2.

Although there is a comprehensive list of repair mechanisms, spermatozoa possess a limited repair repertoire due to their condensed nature, which renders their DNA essentially inert to facilitate a safe union with the oocyte. Mature sperm face significant barriers to utilizing repair mechanisms, given their protamine‐rich DNA and compaction level. During spermatogenesis, DNA damage repair occurs until the third week, after which sperm DNA undergoes significant condensation alongside the downregulation of repair mechanisms.^27^ Despite limitations of DNA repair in mature sperm, studies have shown that spermatozoa can still fertilize oocytes successfully despite DNA fragmentation, likely because oocytes possess robust repair mechanisms.^45^, ^46^, ^47^ While a truncated BER pathway has been observed in spermatozoa through the presence of the enzyme OGG1, responsible for cleaving DNA base adducts, the primary responsibility for reducing the risk of paternally inherited genomic errors lies with the oocyte.^46^ The current accepted paradigm within sperm DNA repair mechanisms post‐fertilization is that the oocyte plays a crucial role in rescuing the sperm's DNA damage. This reliance is supported by the presence of maternal mRNAs and proteins existing in the oocyte before ovulation and the high expression of DNA repair genes in human oocytes.^45^, ^48^, ^49^

### 3.3 Type of SDF testing

SDF has become an intriguing area of research in the context of infertility, serving as a potential biomarker for diagnosing sperm‐related infertility. Several approaches have been developed to assess SDF with direct clinical applications, although they exhibit varying sensitivity and specificity among the different assays. The tests utilized in clinical ART practices include the terminal deoxynucleotidyl transferase dUTP nick end labeling (TUNEL) assay, the sperm chromatin structure assay (SCSA), the sperm chromatin dispersion (SCD) test (also known as the Halo test), and the comet assay, which includes the neutral, alkaline, and two‐tailed (2 T) assay types (Table 1). Another rising area of research in infertility is the immunodetection of γH2AX as a biomarker for DSBs. These tests are either categorized as direct tests which analyze the number of DNA breaks and include the Comet and TUNEL assays or indirect tests which detect the susceptibility of chromatin to acid treatment via the SCSA and SCD assays.^4^, ^50^

**TABLE 1:** Types of assays for sperm DNA fragmentation detection.

Assay	Purpose/intent	Damage detected	Resources needed	Advantages	Disadvantages
TUNEL	Relies on TdT labeling the 3′‐OH free ends of dsDNA directly measuring fragmented sperm cells; labels the breaks present in DNA with nucleotides (typically deoxyuridine triphosphate dUTP) catalyzed by the DNA polymerase TdT	SSB/DSB	Fluorescence microscopy, flow cytometry or light microscopy	Standardized protocol and correlation with other assays, sensitive so can be done on samples with low sperm yields	Requires flow cytometry or optical/fluorescent microscopy, inter‐lab variability
SCSA	Uses AO fluorescence to measure denaturation of the chromatin that signifies DNA breaks	DSB	Flow cytometer	Standardized protocol, fast assay	Need for flow cytometry and trained personnel
SCD/Halo	Sperm with fragmented DNA fails to produce the characteristic dispersion “halo” upon mixture with aqueous agarose following acid and lysing treatments	DSB/SSB	Fluorescence microscope	Rapid and inexpensive assays that are easy to use without need for expensive equipment or trained personnel	Inter‐observer variability
Alkaline Comet	Upon DNA compaction and protein depletion, sperm undergo single‐cell electrophoresis under alkaline conditions with the “Comet” forming toward the cathode end from fragmented DNA	mainly SSB, but also DSB	Electrophoresis and fluorescence microscope	Fast assay, correlation with other assays (SCD, TUNEL, Comet); requires low sperm counts	Technique and analysis not standardized and requires expertise; inter‐observer variability
Neutral Comet	Upon DNA compaction and protein depletion, sperm undergo single‐cell electrophoresis under neutral conditions with the “Comet” forming toward the cathode end from fragmented DNA	DSB	Electrophoresis and fluorescence microscope	Fast assay, requires low sperm counts	Lack of correlation with other assays (SCD, TUNEL, Comet), technique and analysis not standardized and requires expertise, inter‐observer variability
Two‐Tailed Comet (2T‐Comet)	Reveals SSB and DSB in individual cells by subjecting the deproteinized sperm to electrophoresis under denaturing conditions to isolate damaged fragments of DSB following a second electrophoresis running parallel under alkaline conditions to induce denaturation and expose fragmented SSB resulting in a two‐dimensional comet tail emerging from the core where the different strand fragmentation types are simultaneously detected	Distinguishes DSB and SSB	Electrophoresis and fluorescence microscope	Fast assay, requires low sperm counts, differentiates DSB and SSB	Technique not standardized and requires expertise with analysis

Abbreviations: DSB, double‐strand break; SCD, sperm chromatin dispersion; SCSA, sperm chromatin structure assay; SSB, single‐strand break; TUNEL, terminal deoxynucleotidyl transferase dUTP nick end labeling.

The TUNEL assay measures both DSBs and SSBs by utilizing terminal deoxynucleotidyl transferase. This enzyme catalyzes the attachment of deoxynucleotides labeled with fluorochrome or another fluorescent marker to the 3′‐hydroxyl termini of DNA. By labeling spermatozoa with fragmented DNA, both SSB and DSB can be detected.^51^, ^52^ The SCSA evaluates the susceptibility of sperm DNA to denaturation using acridine orange fluorescence to identify DSBs and SSBs.^53^, ^54^ In contrast, the SCD assay relies on microscopic observation of a “halo,” which represents chromatin dispersion following denaturation and deproteinization. Sperm with fragmented DNA do not exhibit this characteristic halo of dispersed DNA loops seen in non‐fragmented DNA, making it useful for detecting both DSBs and SSBs.^51^ The Comet assay involves lysing cells under neutral or alkaline conditions to form deproteinized nuclei, and the resulting DNA is electrophoresed. Fragmented DNA moves toward the anode, creating a visual pattern resembling a comet, with the extent of the tail indicating the level of DNA damage. The neutral comet assay detects DSBs under neutral lysis conditions, while the alkaline comet assay mobilizes DNA under alkaline conditions, detecting both DSBs and SSBs without distinguishing between them.^27^, ^51^, ^55^, ^56^, ^57^ Given the differential repair mechanisms for DSBs and SSBs, it is crucial to distinguish between these types of fragmentation in clinical applications related to ICSI or IVF ART. The 2T comet assay provides a solution to this problem by differentiating the total level of sperm DNA fragmentation (SDF) into SSBs and DSBs in individual cells.^53^ Tests that detect γH2AX, the phosphorylated form of the histone H2AX, immediately following DSBs, have emerged as promising predictors of success with ICSI, but further research involving randomized control trials is needed before they can be applied clinically.^27^, ^58^

While several tests are available for SDF testing, the lack of consistency in assay sensitivity and specificity for clinical applications, along with discrepancies in the literature regarding the overall association between increased SDF and clinical fertility outcomes among different assays, complicate the universal predictive abilities of these tests. The quest to establish a consistent relationship between SDF and fertility outcomes faces challenges due to the lack of a standardized definition and assay to define increased DNA fragmentation index (DFI) in couples experiencing RPL caused by sperm‐related factors. Each assay has its own threshold values to identify clinical SDF cutoffs for predicting pregnancy, leading to variability in results.^50^ A review article on clinical guidelines for SDF compiled different studies that attempted to identify clinical cutoffs for predicting natural or ART pregnancies using these assays. The TUNEL assay showed cutoff values ranging from 4% to 20%, with most values falling in the late teens, while the Comet assay ranged from 44% to 56%. The SCSA results ranged from 10.4% to 30.3%, and the SCD assay ranged from 9.7% to 28.5%, with most values falling in the late 20s. Such differences among assays make it challenging to establish a universal SDF analysis standard.

Several studies have made claims about the predictive value of different assays for IVF and ICSI outcomes, but contradictory results have also been reported. However, there is a lack of comprehensive studies that directly compare the different assays, resulting in a lack of consensus regarding the standard threshold for defining increased DFI across these assays. This inconsistency poses challenges in interpreting the data and limits the universal clinical implementation of these assays for patients with infertility. For instance, a comparative study of the TUNEL, SCSA, and comet assays found weak correlations among the three when analyzed as continuous variables, making it questionable to take these results at face value.^54^ A study by Ribas‐Mayou et al. compared the TUNEL, SCSA, SCD, and different comet assay types within a single population. The results showed statistically significant differences in SDF between fertile and infertile patients using the TUNEL assay, SCSA, SCD test, and alkaline comet assays. However, no differences were found between patients when using the neutral comet assay. Correlation analyses indicated the strongest correlations between the cytometric assays (TUNEL and SCSA), as well as correlations between the SCD test and SCSA, and the SCD test and TUNEL assay. These findings further highlight the complexity in establishing a clear consensus on the parameters for elevated DFI within SDF assessment. The correlation values were relatively lower between the alkaline comet assay and the SCD test, the alkaline comet and SCSA, and the alkaline comet and TUNEL assay, with no significant correlations between the neutral comet assay and the other four methodologies. To assess the clinical utility of different SDF tests for predicting infertility, Ribas‐Maynou et al. also utilized receiver operating characteristic curves to determine sensitivity and specificity. Their results showed the alkaline comet assay as the most predictive method, followed by the TUNEL assay, the SCD test, SCSA, and the neutral comet assay. This study argues that different techniques may measure various aspects of SDF, which supports the claim that SA protocols should incorporate multiple SDF testing methods.^51^

The debate surrounding the clinical implementation of SDF testing is controversial among physicians and professional health organization guidelines. In 2013, the American Society for Reproductive Medicine (ASRM) guideline on the utility of sperm DNA integrity testing concluded that SDF is common in patients experiencing infertility, but the current testing methods lack reliable predictability for routine clinical use.^59^ The ASRM's 2015 guideline for the diagnostic workup of infertile patients acknowledged SDF and its association with spontaneous recurrent miscarriage but highlighted the limited data available to support routine clinical inclusion of abnormal DNA‐integrity assessments in the diagnostic process. The guideline addressed the clinical implications of abnormal SDF on IVF or ICSI outcomes. It emphasized that the available data on the relationship between abnormal DNA integrity and reproductive outcomes are limited, making routine testing currently impractical. The guideline called for further research and clinical studies to identify subgroups of patients who might benefit from SDF testing.^60^ In contrast, the 2020 American Urological Association (AUA)/ASRM guidelines for diagnosing and treating infertile patients did not recommend SDF testing as part of the initial evaluation due to inadequate data to support routine usage. Although the guidelines acknowledged the negative association of DNA fragmentation with pregnancy rates and its positive association with miscarriages, they mentioned that the definition of high levels of SDF remains unclear. Medical associations advised counseling infertile patients about the potential association of high SDF with miscarriage and considered SDF testing for couples experiencing RPL because sperm from patients with RPL may have higher SDF levels.^50^ The 6th Edition of the WHO manual for laboratory semen testing, published in 2021, includes a dedicated section to SDF and highlights how meta‐analyses have suggested that SDF may affect embryo development, implantation, and natural and assisted pregnancies. The manual outlines the protocols for conducting the TUNEL assay, SCD test, Comet assay, and SCSA assay.^4^ The European Association of Urology's 2021 guidelines for sexual and reproductive health recommend performing SDF testing in the evaluation of couples experiencing RPL and unexplained infertility.^15^ However, various clinical guidelines have expressed differing opinions on the routine use of SDF testing in fertility care.^18^, ^61^, ^62^, ^63^ The complexity of infertility, which involves multiple contributing factors between couples, makes it challenging to attribute SDF as the sole reason for the need for ART. Therefore, incorporating SDF testing into clinical and professional health organization guidelines presents difficulties.

### 3.4 Effect of SDF on fertility outcomes

Over the last two to three decades, numerous studies, including clinical trials, have been conducted to investigate the impact of SDF on fertility outcomes. Many systematic reviews and meta‐analyses, particularly ART settings, have also been conducted to summarize its effects.^64^ Most of the meta‐analyses have shown significant adverse effect of high SDF on fertility outcomes. However, the significance of SDF in clinical practice is still controversial and the evaluation of SDF is not recommended for routine initial evaluation of AMAB partners.^17^ This is mainly due to the studies analyzed by meta‐analyses being cohort studies and lacked randomization. This truth results in weak connection of SDF on initial management of infertile couples. Oocytes are known to play an important role in repairing sperm DNA damage.^65^ Yet, its capacity depends on assigned female at birth (AFAB) individuals as the extent of repair mechanism contribution and the threshold of sperm DFI at which an oocyte can repair such damage are unclear. The oocytes' mechanistic involvement further contributes to complexity in evaluating the effect of SDF in infertility. In the fertilization and embryo development process, the paternal gene derived from spermatozoa will be activated after zygotic transcriptional activation, known as late paternal effect.^66^ The impact of high SDF on the reproductive process is more apparent after the 4‐ to 8‐cell embryo stage rather than during fertilization. This is supported by studies showing that high SDF negatively affects embryo development but not fertilization outcomes.^67^, ^68^ On the other hand, the effect of SDF on pregnancies or live births, both in natural pregnancies and ART settings, are more heterogeneous. This heterogeneity is partly due to confounding AFAB factors that are challenging to adjust and account for in research settings.

Although there are limitations and difficulties in previously conducted studies as described above, several systematic reviews and meta‐analyses have demonstrated that high SDF adversely affects pregnancy rates in spontaneous pregnancy, intrauterine insemination (IUI), and IVF.^69^, ^70^, ^71^, ^72^, ^73^ In contrast, for ICSI, the majority of meta‐analyses have not shown a significant effect of SDF on pregnancy rates.^72^, ^74^, ^75^, ^76^ Thus, the impact of SDF appears lower in studies of ICSI compared to those of IVF, despite the fact that they both needs in vitro culture and implantation of embryos. The reason for this is not fully understood. One possible reason is that gametes in ICSI have less exposure to the surrounding environment in prolonged culture as opposed to with IVF. This may result in less sperm DNA damage at the time of fertilization.^77^ The effect of SDF on live birth rates is more variable, with recent meta‐analyses showing no significant relationship between high SDF and live birth rates,^76^ while previous large‐scale meta‐analyses reported significantly reduced live birth rates in the high SDF group after both IVF and ICSI.^78^

#### 3.4.1 Spontaneous pregnancy

Zini conducted a meta‐analysis that included three studies^79^, ^80^, ^81^ with a total of 616 cases. The analysis revealed a significant correlation between sperm DNA damage and the failure to achieve a natural pregnancy (odds ratio: 7.01, 95% confidence interval [CI] 3.68–13.36, *p* < 0.001).^69^ Although the effect of conventional parameters such as concentration or motility was not corrected in the meta‐analysis, SDF was proven as an independent factor for infertility by multivariate regression or other analysis in included studies. However, there have been only limited studies conducted to investigate whether SDF can predict the likelihood of natural pregnancy. Additionally, the effect of SDF on live birth rate was not examined in this study. To obtain more robust evidence in the relation between SDF and these outcomes, large‐scale prospective cohort studies designed with low risk of bias are needed. Studying spontaneous pregnancies presents challenges due to the high number of confounding factors that can influence the outcomes.

#### 3.4.2 IUI

Three meta‐analyses have investigated the association between high SDF and IUI outcomes.^69^, ^71^, ^82^ Zini et al. evaluated one valid article^83^ and found that high SDF was associated with lower IUI pregnancy rates (OR: 9.9, 95%CI: 2.37–41.51, *p* < 0.0001).^69^ Sugihara et al. included three studies comprising 917 IUI cycles and reported that low SDF was associated with a higher clinical pregnancy rate (RR: 3.30, 95%CI: 1.16–9.39).^71^ Chen et al. analyzed 10 studies involving 2839 cycles and found a significant association between high SDF and low pregnancy rate (RR: 0.34, 95% CI; 0.22–0.52, *p* < 0.001), as well as low delivery rate of IUI (RR: 0.14, 95% CI; 0.04–0.56, *p* < 0.001).^82^ These findings collectively indicate that high SDF is linked to reduced success rates in IUI.

#### 3.4.3 IVF and ICSI

##### Fertilization and embryo quality

Regarding the fertilization rate, Chen et al. conducted a meta‐analysis that included 12 articles.^84^ They found that there was no statistically significant association between high SDF and fertilization rate, both in IVF (RR = 0.94, 95% CI 0.77–1.14, *p* = 0.61) and ICSI (RR = 0.79, 95% CI 0.52–1.18, *p* = 0.25). Li et al. also reported similar results, showing no significant association between high SDF and fertilization rate in both IVF and ICSI.^70^ The association between SDF and embryo quality has been the subject of several meta‐analyses and reviews.^72^, ^74^, ^85^ Simon et al. reviewed 80 articles comprising 14 871 treatment cycles^85^ and found that 27 out of 80 (34%) studies reported a significant association between higher SDF and lower embryo quality. Zini conducted a systematic review and found that 11 out of 28 studies reported a significant association between sperm DNA damage and low embryo quality.^74^ Although these reviews seem to show conflicting effects of SDF on embryo development, Deng et al. conducted a meta‐analysis on 8 studies involving 17 879 embryos. They found that embryos with high DFI had a lower rate of good‐quality embryos compared to those with low DNA fragmentation (42.8% vs. 69.7%, RR = 0.65 [0.62, 0.68], *p* < 0.01).^72^ This suggests that high SDF is indeed associated with lower embryo quality, and the lack of significant association between SDF and fertilization rates may be attributed to the late paternal effect.

##### Implantation rate/ clinical pregnancy rate

Ribas‐Maynou et al. conducted a meta‐analysis and reported that high SDF had a negative effect on IVF, with a significant impact on implantation (RR = 0.68; 0.52–0.89; *p* < 0.01) and pregnancy rates (RR = 0.72, 95%CI:0.55–0.95, *p* = 0.02). However, the latter effect turned out to be not statistically significant after adjusting for publication bias. In contrast, implantation and pregnancy rates with ICSI were not significantly affected by SDF.^76^

The majority of the previous studies have shown a significant association between high DFI and low pregnancy rates after IVF.^69^, ^70^, ^72^, ^73^, ^75^, ^76^ Zhao et al. reviewed 9 studies comprising 1268 IVF cycles and found a significant decrease in pregnancy rate in patients with high sperm DNA damage (OR = 0.66, 95% CI: 0.48–0.90, *p* = 0.008).^75^ Deng et al. also reported that the clinical pregnancy rate was significantly lower in the high DFI than in the low DFI (OR = 1.92, 95%CI: 1.33–2.77, *p* = 0.0005) by evaluating 2130 IVF cycles from 7 studies.^72^ However, the pregnancy rate after ICSI has been shown to have an insignificant association with SDF in most meta‐analyses.^69^, ^70^, ^72^, ^73^, ^75^, ^76^ Only the meta‐analysis conducted by Simon showed a statistically significant impact of SDF on clinical pregnancy after ICSI,^73^ while the other five meta‐analyses have concluded that the association was statistically insignificant.

This difference in the impact of SDF between IVF and ICSI may be attributed to the difference in their procedures. In ICSI, spermatozoa are manually injected into the oocyte, bypassing the random fertilization process. Conversely, in IVF, spermatozoa are co‐incubated with oocytes, and fertilization occurs randomly as spermatozoa penetrate the zona of the oocyte. During this process, natural sperm selection can occur, where spermatozoa with high SDF might be naturally excluded. In IVF, however, the gametes are subjected to prolonged culture during the fertilization process. Thus, spermatozoa may be damaged by the surrounding environment such as ROS in culture media, Conversely, spermatozoa are injected into the oocyte within a few hours of ejaculation in ICSI and they can avoid the exposure.^86^ Another possibility is that bias between patient populations seeking these ARTs. Patients undergoing ICSI tend to have worse clinical conditions that are independent of SDF. It is difficult to eliminate all these bias factors, even if adjustment analyses are incorporated.

##### Live birth rate

Although the most important outcome for infertile couples is live birth rate, only a few studies have evaluated this as an outcome, and each study had a relatively small sample size. Two available meta‐analyses have evaluated live birth rates.^76^, ^78^ Osman et al. found a significant association between low SDF levels and high live birth rates after both IVF (RR = 1.27, 95%CI: 1.05–1.52, *p* = 0.01) and ICSI (RR = 1.11, 95%CI: 1.00–1.23, *p* = 0.04).^78^ However, after sensitivity analysis with adjustments, the significance was not observed in ICSI (RR = 1.08, *p* = 0.88). In contrast, Ribas‐Maynou et al. reported that SDF did not have a significant effect on either IVF (RR = 0.48, 95%CI: 0.22–1.02, *p* = 0.06) or ICSI (RR = 0.92, 95%CI: 0.67–1.27, *p* = 0.62).^76^ Similarly, Deng et al. also found no significant effect of SDF on live birth rates after combined IVF and ICSI.^72^

#### 3.4.4 Recurrent pregnancy loss

RPL has posed significant challenges in the field of reproductive medicine, and while various factors are believed to contribute to its occurrence, the underlying mechanism is not fully understood.^87^ In addition to the well‐studied maternal causes, there is a growing body of evidence highlighting the impact of AMAB factors, including SDF. Recent animal studies have demonstrated that sperm DNA damage can lead to the fragmentation and random distribution of paternal chromosome segments in developing embryos, resulting in chaotic mosaicism with non‐identical genotypes in cells.^88^ Although the oocyte can repair some sperm DNA damage after fertilization, high levels of damage beyond the oocyte's repair capacity can lead to poor embryo development, implantation failure, and miscarriage.^47^, ^89^ In a meta‐analysis conducted by Robinson et al. in 2012, involving 2969 couples, it was demonstrated that SDF is associated with sporadic miscarriage (RR = 2.16, 95%CI: 1.54–3.03, *p* < 0.00001).^90^ In the context of RPL, a recent meta‐analysis by McQueen et al. also showed that the partners of patients with a history of RPL have a significantly higher rate of SDF compared to the partners of fertile control patients without RPL (mean difference of 10.7%, 95% CI: 5.82–15.58, *p* < 0.0001).^91^ Considering the high risk of miscarriage associated with abnormal SDF, the latest ASRM guidelines now recommend evaluating SDF in addition to karyotype analysis for couples experiencing RPL.^17^ Similarly, the 2022 ESHRE guideline for RPL also acknowledges the importance of assessing SDF in such couples for diagnostic purposes, not solely for explanatory purposes, based on the emerging evidence from recent analyses.^87^

The compiled meta‐analyses on SDF based on the type of conception and their effects are presented in Table 2. Overall, previous studies have shown the potential of SDF as a predictor of reproductive outcomes, particularly in ART. Evaluating SDF may aid clinicians in making decisions about whether to proceed with ICSI for patients with high SDF, where the impact of SDF on outcomes is minimal or insignificant. However, it should be noted that the role of SDF as a predictor in reproductive outcomes is still not definitively established. Some original studies have reported no significant association between SDF and reproductive outcomes.^92^, ^93^ The overall impact of meta‐analyses is also limited by the scarcity of well‐conducted RCTs in previous studies. As discussed earlier, patient cohorts in clinical conditions may introduce biases, and the number of non‐adjustable confounding factors increases when evaluating final outcomes such as live birth rates. In addition, there is heterogeneity in the assays used for SDF measurement and the determination of cutoff values. Thus, standardizing the assays and cutoff values is crucial for establishing SDF as a more robust marker in future clinical practice.

**TABLE 2:** Literature of meta‐analyses on sperm DNA fragmentation and reproductive outcomes.

Conception	Outcome	Sample size	SDF assays used in evaluated studies	Results	Author, year
Spontaneous Pregnancy	Pregnancy	3 studies, 616 cases	SCSA	Correlation between sperm DNA damage and failure to achieve a natural pregnancy (OR = 7.01, 95%CI:3.68–13.36, *p* < 0.001)	Zini, 2011^69^
IUI	Pregnancy	1 study, 387 cycles	SCSA	Association between high SDF with lower IUI pregnancy rates (OR = 9.9, 95%CI:2.37–41.51, *p* < 0.0001)	Zini, 2011^69^
		3 studies, 917 cycles	SCSA, SCD	Low SDF associated with higher clinical pregnancy rate (RR = 3.30, 95%CI:1.16–9.39)	Sugihara, 2020^71^
		10 studies, 2839 cycles	SCSA, SCD	Significant association between high SDF and lower pregnancy rate (RR = 0.34, 95%CI:0.22–0.52, *p* < 0.001)	Chen, 2019^81^
	Live Birth	2 studies, 518 cycles	SCSA	Significant association between high SDF and lower live birth rate (RR = 0.14, 95% CI; 0.04–0.56, *p* < 0.001)	Chen, 2019^81^
IVF	Fertilization	4 studies, 770 cycles	TUNEL, SCSA	No significant association between high SDF and fertilization rate (RR = 0.79, 95% CI 0.54 to 1.16, *p* = 0.23)	Li, 2006^70^
		5 studies, 1499 cycles	SCSA	No significant association between high SDF and fertilization rate (RR = 0.94, 95% CI 0.77–1.14, *p* = 0.61)	Chen, 2022^83^
	Pregnancy	5 studies, 816 cycles	TUNEL, SCSA	Clinical pregnancy rate decreased significantly for patients with high SDF (RR = 0.68, 95%CI:0.54–0.85, *p* = 0.006)	Li, 2006^70^
		9 studies, 1268 cycles	TUNEL, SCSA, Comet	Significant decrease in pregnancy in patients with high sperm DNA damage (OR = 0.66 95%CI:0.48–0.90, *p* = 0.008)	Zhao, 2014^74^
		11 studies, 1805 cycles	TUNEL, SCSA, CC, CMA3	Sperm DNA damage is associated with significant reduction in IVF pregnancy rates (OR = 1.70, 95%CI:1.30–2.23, *p* < 0.05)	Zini, 2011^69^
		16 studies, 3734 cycles	TUNEL, SCSA, SCD, Comet	Significant negative effect of SDF on clinical pregnancy after IVF (OR = 1.92, 95%CI:1.33–2.77, *p* = 0.0005)	Simon, 2017^85^
		7 studies, 2130 cycles	SCSA, SCD, Comet	The clinical pregnancy rate was significantly lower in the high DFI than in the low DFI (RR = 0.77, 95%CI:0.59–1.00, *p* = 0.05)	Deng, 2019^72^
		15 studies, 3711 cycles	TUNEL, SCSA, SCD, Comet	Significant negative association between sperm DNA damage and pregnancy rate (RR = 0.72, 95%CI:0.55–0.95, *p* = 0.02)	Ribas‐Maynou, 2021^75^
	Live birth	4 studies, 553 cases	SCSA, TUNEL, Comet	Significantly higher live birth rate after IVF in men with low SDF (RR = 1.27, 95%CI:1.05–1.52, *p* = 0.01)	Osman, 2015^77^
		6 studies, 1634 cycles	TUNEL, SCSA, SCD, Comet	No significant relationship between DNA damage and live‐birth rate was observed (RR = 0.48, 95%CI:0.22–1.02, *p* = 0.06)	Ribas‐Maynou, 2021^75^
ICSI	Fertilization	3 studies, 201 cycles	TUNEL, SCSA	No significant difference in fertilization rate between high and low SDF (RR = 1.03, 95%CI:0.89–1.18, *p* = 0.70)	Li, 2006^70^
		5 studies, 1893 cycles	SCSA	No significant statistically between high SDF and fertilization rate (RR = 0.79, 95%CI:0.52–1.18, *p* = 0.25)	Chen, 2022^83^
	Pregnancy	3 studies, 201 cycles	TUNEL, SCSA	No significant difference in clinical pregnancy rate between high and low SDF (RR = 0.76, 95%CI:0.55–1.04, *p* = 0.09)	Li, 2006^70^
		10 studies, 1047 cycles	TUNEL, SCSA, Comet, AO	No significant decrease in pregnancy in patients with high DNA damage (OR = 0.94, 95%CI:0.70–1.25, *p* = 0.65)	Zhao, 2014^74^
		14 studies, 1171 cycles	TUNEL, SCSA, Aniline Blue, CMA3	Sperm DNA damage is not associated with lower pregnancy rate in ICSI (OR = 1.15, 95%CI:0.90–1.55, *p* = 0.65)	Zini, 2011^69^
		24 studies, 2282 cycles	TUNEL, SCSA, SCD, Comet	Significant negative effect of SDF on clinical pregnancy after ICSI (OR = 1.49, 95%CI:1.11–2.01, *p* = 0.0075)	Simon, 2017^85^
		4 studies, 278 cycles	SCSA, SCD	No significant differences in clinical pregnancy rate between high and low DFI (RR = 0.75 95%CI:0.44–1.27, *p* = 0.29)	Deng, 2019^72^
		25 studies, 5467 cycles	TUNEL, SCSA, SCD, Comet	The association between sperm DNA damage and pregnancy rate not significant but showed a tendency toward a negative effect (RR = 0.89, 95%CI:0.78–1.02, *p* = 0.09)	Ribas‐Maynou, 2021^75^
	Live birth	5 studies, 445 cases	SCSA, TUNEL, Comet	Higher live birth rate after ICSI in men with low SDF (RR 1.11, 95% CI 1.00 to 1.23, *p* = 0.04). No significant difference was confirmed after sensitivity analysis (*p* = 0.88)	Osman, 2015^77^
		9 studies, 3017 cycles	TUNEL, SCSA, SCD, Comet	No significant relationship between DNA damage and live‐birth rate (RR = 0.92, 95%CI:0.67–1.27, *p* = 0.62)	Ribas‐Maynou, 2021^75^
IVF or ICSI	Embryo quality	8 studies, 17 879 embryos (IVF or ICSI)	SCSA, SCD, Comet	Lower good‐quality embryo rate with high DFI group (42.8% vs. 69.7%) (RR = 0.65, 95%CI:0.62–0.68, *p* < 0.01)	Deng, 2019^72^

Abbreviations: CC indicates chromatin compaction; CMA3, chromomycin A3; DFI, DNA fragmentation index; ICSI, intracytoplasmic injection; IVF, in vitro fertilization; IUI, intrauterine insemination; OR, odds ratio; RR, relative risk; SCD, sperm chromatin dispersion; SCSA, sperm chromatin structure assay; SDF, sperm DNA fragmentation; TUNEL, terminal deoxynucleotidyl transferase (TdT) dUTP Nick‐End Labeling.

### 3.5 SDF and cost

The estimated cost of each SDF testing kit ranges between $175–400 in the United States.^94^ SDF testing is usually not covered by medical insurance alongside other fertility‐related therapeutic interventions.^95^ In a case scenario with high SDF, it will cost more than $1000 for the SDF testing. Additional costs for main treatment to improve SDF include medication and varicocelectomy. Moreover, equipment and running cost also need to be considered by providers. Some of the assays such as TUNEL and SCSA need flow cytometry, which remains one of the most expensive pieces of laboratory equipment. The cost varies, but it typically ranges from $100 000–$500 000 in the United States. Running flow cytometry requires well‐trained technicians, thus an additional barrier and cost. Although the cost of SDF testing is currently one of the important factors limiting its routine use in clinical practice, appropriate performance of SDF testing should ideally reduce the overall treatment cost by assisting robust clinical fertility decision‐making process.

### 3.6 How to manage high SDF?

#### 3.6.1 Lifestyle changes

High levels of SDF have been associated with various lifestyle factors that may negatively impact overall health. These include metabolic conditions such as diabetes mellitus, obesity, and tobacco smoking. A recent meta‐analysis by Szabo et al. revealed that impaired glycemic control and smoking are associated with higher levels of SDF.^13^ This suggests that adopting a healthy lifestyle, which includes a balanced diet, regular exercise, and refraining from smoking, may help reduce or prevent high levels of SDF.^96^ Specifically, engaging in high‐intensity exercises, following a diet rich in vegetables, fish, seafood, and whole grains (e.g., the Mediterranean diet), and reducing alcohol intake may have favorable effects on SDF.^97^ Cigarette smoking in particular has been strongly associated with high SDF. Studies have consistently demonstrated that smokers tend to have significantly higher levels of SDF when compared to non‐smokers.^97^, ^98^ The exact mechanism behind this association is likely complex and involves factors such as higher levels of ROS and apoptotic caspases in infertile smokers. Additionally, gene variations induced by smoking and exposure to toxic polycyclic aromatic hydrocarbons present in cigarettes may play a role.^99^, ^100^, ^101^ Overall, these studies emphasize the importance of quitting or reducing cigarette smoking to prevent higher SDF levels, which could have a significant impact on fertility and overall reproductive health.

#### 3.6.2 Varicocele repair

Varicocele is the second most common cause of AMAB infertility after idiopathic infertility, affecting approximately 35%–44% of infertile AMAB patients.^102^ The condition is characterized by reflux in the spermatic vein, leading to increased temperature in scrotum, which in turn degrades spermatogenesis. Moreover, the testicular microenvironment experiences reduced oxygen levels due to the inefficient venous return, resulting in heat stress and oxidative stress that adversely affect AMAB fertility and contribute to SDF.^103^ As a result, patients with clinical varicocele tend to have higher SDF levels compared to controls.^104^ Fortunately, varicocele repair has been found to significantly reduce SDF levels in patients with clinical varicocele, as reported in recent meta‐analyses.^105^, ^106^ Moreover, studies have indicated that varicocele repair in infertile patients with clinical varicocele and high SDF can lead to significant improvements in clinical pregnancy and live birth rate after ICSI.^107^ Thus, varicocele repair is recommended for infertile AMAB patients with clinical varicocele and high SDF. However, there has been some debate about whether varicocele repair should be offered to patients with otherwise normal semen parameters but high SDF.^108^ A recent global survey on varicocele management showed that 34.7% of reproductive experts consider varicocele repair for patients with a history of infertility and increased SDF, even if conventional semen parameters are normal. Nonetheless, further well‐designed studies are needed to establish the efficacy of varicocele repair in such cases.^109^

#### 3.6.3 Role of antioxidants

The potential of antioxidant treatment to mitigate the deleterious impact of ROS on SDF has been explored as a promising approach to SDF management. Superoxide dismutase supplementation (SOD) has been studied in various centers and has been found to be independently associated with improvements in SDF according to multivariate analysis.^110^, ^111^ However, the results may be limited by the fact that some studies were conducted at single centers or retrospective in nature. Meta‐analyses of RCTs have demonstrated a significant reduction in SDF compared to a placebo,^14^ specifically with the use of butylated hydroxytoluene (BHT), vitamin E, and tempol.^112^ Nonetheless, the optimal dosing, regimen length, and timing for antioxidant therapy have not been fully established, and certain studies have suggested no significant difference in SDF for otherwise healthy, AMAB infertile patients.^113^ The actual impact of antioxidant supplementation for AMAB subfertility on pregnancy rates and live birth has been a big controversial topic. Although the latest Cochrane Review has shown that antioxidant supplementation may increase clinical pregnancy rates (OR = 1.89) and change of live birth (OR = 1.43), the analysis has concluded that the overall certainty of evidence was very low from small number of RCTs.^114^ The Males, Antioxidants, and Infertility (MOXI) randomized controlled trial as a well‐known RCT demonstrated that plasma antioxidant levels at baseline were not predictive of successful pregnancy or live birth.^115^ Additionally, antioxidant levels no statistically significant correlation between‐at baseline or following 3 months of treatment‐ antioxidant levels (specifically vitamin E, selenium, and zinc) and SDF.^115^ Although MOXI trial is an important RCT in this topic, there is argument because of inherent shortcomings in the study design. There is a criticism that this study was ended after the pilot phase of 3 months completed before it reached decent power size.^116^ Larger RCT with robust methodology needs to be conducted to provide further conclusions. Overall, oral antioxidant therapy may be a cost‐effective option for patients with elevated SDF; however, the selection of treatment should be guided by individual decisions made between the healthcare professional and the patient. It is also essential to consider any potential adverse effects of supplementation. It is crucial to note that antioxidants are not approved by the government as a standard treatment for infertility.

#### 3.6.4 Role of hormonal versus non‐hormonal therapy

Studies have shown that AMAB infertility is associated with lower serum testosterone levels and reduced testosterone‐to‐estradiol ratios.^117^ As a result, several studies have focused on therapies that can modulate the hypothalamic–pituitary‐gonadal axis and/or the peripheral conversion of hormones to explore their impact on SDF. Some evidence suggests that letrozole (a peripheral aromatase inhibitor) or direct administration of follicle‐stimulating hormone (FSH) can significantly reduce SDF and contribute to successful intrauterine pregnancy.^117^, ^118^ Age, baseline testosterone levels, and testicular volume may be predictors of response to FSH administration.^117^, ^118^ Regarding treatment regimens, there is currently no definitive evidence to guide specific paradigms. However, some data suggests that sequential treatment with human chorionic gonadotropin (hCG) and FSH could be effective due to the luteinizing hormone (LH)‐like effect of hCG.^119^ Antioxidant therapy, as discussed earlier, is a non‐hormonal approach for managing elevated SDF. In addition, evidence suggests that addressing infectious diseases may play a crucial role in reducing high SDF. For instance, combination antiretroviral therapy has been found to decrease SDF in HIV patients, and AMAB individuals with human papillomavirus (HPV) infections tend to have higher levels of SDF.^120^, ^121^ This suggests that timely vaccinations and standard interventions to reduce viral load could be beneficial in reducing or preventing high SDF.

#### 3.6.5 Testicular versus ejaculate sperm

Testicular sperm has been found to have lower levels of oxidative damage, potentially leading to decreased SDF and increased fertility rates compared to ejaculated sperm.^122^ However, SDF testing in testicular sperm lacks standardization. Obtaining testicular sperm also requires surgical intervention, which carries some common surgical risks (e.g., surgical site infection, testicular swelling, damage to surrounding structures, etc.), although these complications are rare.^122^, ^123^ Despite these risks, critical analyses of meta‐analyses and primary studies comparing testicular and ejaculated sperm have demonstrated higher pregnancy and live birth rates in patients with high SDF using testicular sperm.^124^, ^125^ Robust RCTs are still lacking, leading the European Association of Urology to refrain from recommending routine testicular sperm retrieval for infertile AMAB patients with high SDF without a full explanation of the risks and the lack of current RCT‐derived data to patients.^125^ Interestingly, a recent survey among global sexual health experts has revealed that a significant portion of urologists might recommend testicular sperm retrieval in select cases.^126^ Thus, the recommendations regarding testicular sperm retrieval for AMAB patients with high SDF remain controversial and unresolved.

#### 3.6.6 Short abstinence interval

Although WHO manual recommends 2–7 days of abstinence for semen analysis to standardize the methodology, an even shorter abstinence interval is reported to be effective in improving semen parameters and SDF.^127^ In the absence of ejaculation, spermatozoa accumulate in the epididymis. In the abstinence interval, spermatozoa is exposed to the damaging of ROS generated by abnormal spermatozoa and granulocytes which results in high SDF.^128^ The mechanism of improvement in semen quality in second ejaculation has not been fully unveiled, but it is hypothesized that shorter epididymal transit time can reduce exposure to ROS, and also cause biochemical changes in consecutive ejaculations, which results in reducing sperm damage.^129^ Although current guidelines do not state the efficacy of shorter abstinence, recent systematic meta‐analysis has shown that a shorter abstinence interval was associated with better semen motility and morphology, and lower SDF.^130^ A systematic review by Ayad et al. showed that shorter abstinence decreased the semen volume and concentration while it increased sperm motility significantly.^131^ Studies have also been focusing on very short abstinence which means second ejaculation less than 3 h after the first on the same day. A study in Italy compared semen parameters from normozoospermic and oligoasthenoteratozoospermia (OAT) AMAB individuals after a 2‐to‐7‐day abstinence period and a one‐hour abstinence period. Both groups revealed a significant improvement in normal morphology and a decrease in SDF with a shorter abstinence period, and only OAT group demonstrated an improvement in motility.^132^ Recent meta‐analysis from the same group conducted by Barbagallo et al. showed that sperm concentration and motility were significantly increased in second ejaculate of OAT patients. In contrast, semen volume and SDF was significantly decreased in the second ejaculate.^133^ Especially in ART settings, the sperm quality is more important than the number of spermatozoa as infertile couples do not need so much total sperm count as needed in spontaneous pregnancy or IUI. Therefore, these findings may suggest that OAT patients undergoing ART may have more benefit from a very short abstinence period. Overall, short abstinence interval seems a simple and useful strategy for obtaining spermatozoa with lower SDF and better quality. More robust RCTs are needed to establish strong recommendations.

#### 3.6.7 Sperm selection in high SDF

**FIGURE 3:**
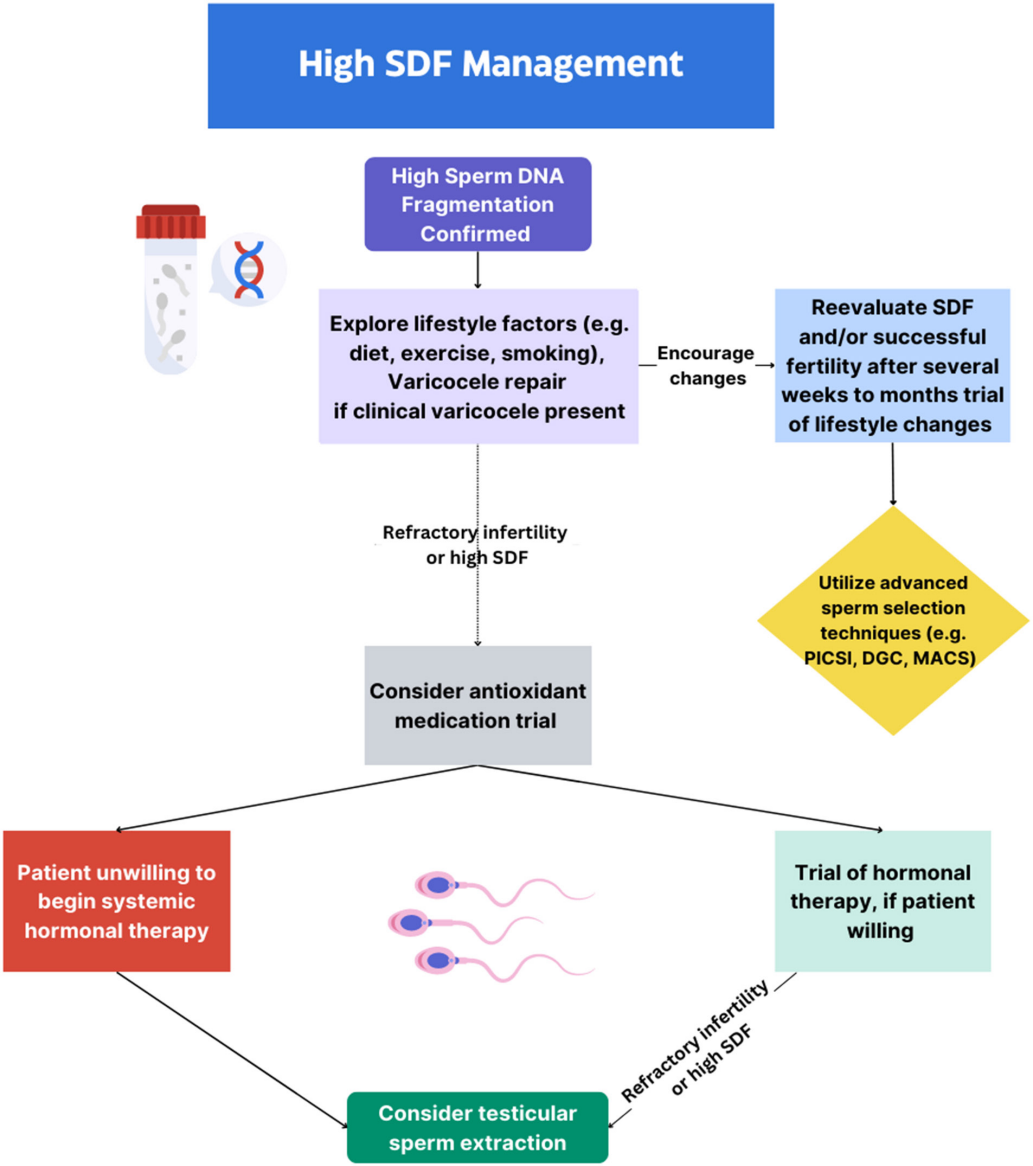
Flowchart illustrating the management for cases with high sperm DNA fragmentation.

Finally, various methods for sperm selection have been explored to improve infertility rates in AMAB patients with high SDF undergoing assisted reproductive techniques. Initial studies on intracytoplasmic morphologically selected sperm injection (IMSI), which involves selecting sperm based on high magnification analysis of sperm morphology, did not show significantly improved clinical outcomes compared to ICSI.^134^ However, later RCTs comparing common sperm selection techniques, such as density gradient centrifugation (DGC), physiologic ICSI (PICSI), and magnetic‐activated cell sorting (MACS), revealed that combining PICSI and MACS with DGC led to significantly higher implementation and pregnancy rates compared to using DGC alone.^135^ Briefly, PICSI involves placing sperm on hyaluronan microdots to mimic high DNA‐integrity binding, leading to the selection of high‐integrity sperm, whereas traditional ICSI relies only on morphology and motility for sperm selection. Conversely, MACS separates apoptosed sperm cells by recognizing externalized phosphatidylserine residues.^135^, ^136^ MACS may be particularly suitable for certain patient populations, as a study by González‐Ravina and colleagues demonstrated comparable gestation, live birth, or miscarriage rates between MACs after DGC and DGC alone for couples undergoing donor IUI (D‐IUI).^136^ Although larger sample sizes and additional multicenter studies are necessary to determine the most effective sperm selection treatments for individuals with SDF, healthcare professionals can discuss selection techniques with patients while acknowledging that robust studies demonstrating the superiority of one technique over another are currently lacking.^126^ The management flowchart for cases with high sperm DNA fragmentation is shown in Figure 3.

## 4 CONCLUSION

This narrative review has presented the latest research articles covering various aspects of SDF, including its mechanism, sperm DNA repair, evidence regarding its impact on reproductive outcomes, and its management. While the efficacy of SDF as a marker has been demonstrated, its application is currently limited to specific situations. For decades, many sperm functional tests have been established in the research field. Within the field of AMAB factor infertility, conventional SA has been the standard routine marker in clinical practice, but it has its limitations. These include poor repeatability and inability to fully assess the fertility of patients. Therefore, there is a pressing need for more reliable markers to evaluate sperm quality, and SDF shows promise as a potential marker for the future. At present, global guidelines recommend SDF evaluation for couples with idiopathic AMAB factors and RPL. High SDF has been associated with lower pregnancy rates in IVF, but its impact on ICSI outcomes remains less clear. In couples experiencing recurrent failure in ART with the AMAB partner showing high SDF, testicular sperm extraction could be considered as an option. For varicocele patients with a history of infertility and normal semen parameters, SDF can help determine the application of varicocelectomy. To establish the true efficacy of SDF in real‐world scenarios, higher‐quality clinical trials, especially RCTs, are necessary. Addressing the variations in cutoff values and subjectivity in SDF assays is crucial for its broader application. Artificial intelligence (AI) could play a significant role in this regard, as recent studies combining SDF techniques with AI have shown promising results.^137^, ^138^ Such attempts may reduce human effort and minimize the subjectivity and inter‐variability in assays, which have been obstacles in determining the efficacy of SDF.

In conclusion, despite its limitations, SDF holds potential for greater inclusion in clinical guidelines. High‐quality clinical trials are essential for standardizing the choice of SDF assays and determine the appropriate DFI cutoff values. Additionally, advancements in assays, including improvements in complexity, subjectivity, and cost‐effectiveness will enhance accessibility to SDF testing. While SDF is perhaps the best‐studied nontraditional sperm test currently available, even this robust set of literature remains contentious, and SDF likely plays a modest role only for a subset of AMAB individuals with infertility. Nevertheless, SDF represents an excellent paradigm and can certainly act as a proof‐of‐principle for the field of andrology to explore other novel sperm tests and expand our knowledge of the diagnosis and treatment of idiopathic AMAB infertility.

## CONFLICT OF INTEREST STATEMENT

Authors declare no Conflict of Interests for this article.

## HUMAN/ANIMAL RIGHTS STATEMENT

This article does not contain any studies involving human or animal subjects.

## Data Availability

The data that support the findings of this study are available from the corresponding author, SK, upon reasonable request.

## References

[1] Vander Borght M , Wyns C . Fertility and infertility: definition and epidemiology. Clin Biochem. 2018;62:2–10. 29555319 10.1016/j.clinbiochem.2018.03.012

[2] Agarwal A , Baskaran S , Parekh N , Cho C‐L , Henkel R , Vij S , et al. Male infertility. Lancet. 2021;397(10271):319–333. 33308486 10.1016/S0140-6736(20)32667-2

[3] Liu Y , Gao J . Reproductive aging: biological pathways and potential interventive strategies. J Genet Genomics. 2023 Mar;50(3):141–150. 35840100 10.1016/j.jgg.2022.07.002

[4] World Health Organization . WHO laboratory manual for the examination and processing of human semen 6th edition. 2021.

[5] Oehninger S , Ombelet W . Limits of current male fertility testing. Fertil Steril. 2019;111(5):835–841. 30975387 10.1016/j.fertnstert.2019.03.005

[6] Wang C , Swerdloff RS . Limitations of semen analysis as a test of male fertility and anticipated needs from newer tests. Fertil Steril. 2014;102(6):1502–1507. 25458617 10.1016/j.fertnstert.2014.10.021 PMC4254491

[7] Niederberger CS . Semen and the curse of cutoffs. J Urol. 2011;185(2):381–382. 21168161 10.1016/j.juro.2010.11.018

[8] Buck Louis GM , Sundaram R , Schisterman EF , Sweeney A , Lynch CD , Kim S , et al. Semen quality and time to pregnancy: the longitudinal investigation of fertility and the environment study. Fertil Steril. 2014;101(2):453–462. 24239161 10.1016/j.fertnstert.2013.10.022 PMC3946620

[9] Boeri L , Belladelli F , Capogrosso P , Cazzaniga W , Candela L , Pozzi E , et al. Normal sperm parameters per se do not reliably account for fertility: a case‐control study in the real‐life setting. Andrologia. 2021;53(1):e13861. 33125742 10.1111/and.13861

[10] Hamada A , Esteves SC , Nizza M , Agarwal A . Unexplained male infertility: diagnosis and management. Int Braz J Urol. 2012;38(5):576–594. 23131516 10.1590/s1677-55382012000500002

[11] García‐Rodríguez A , Gosálvez J , Agarwal A , Roy R , Johnston S . DNA damage and repair in human reproductive cells. Int J Mol Sci. 2018;20(1):31. 10.3390/ijms20010031 30577615 PMC6337641

[12] Pagliuca C , Cariati F , Bagnulo F , Scaglione E , Carotenuto C , Farina F , et al. Microbiological evaluation and sperm DNA fragmentation in semen samples of patients undergoing fertility investigation. Genes. 2021;12(5):654. 10.3390/genes12050654 33925640 PMC8145398

[13] Szabó A , Váncsa S , Hegyi P , Váradi A , Forintos A , Filipov T , et al. Lifestyle‐, environmental‐, and additional health factors associated with an increased sperm DNA fragmentation: a systematic review and meta‐analysis. Reprod Biol Endocrinol. 2023;21(1):5. 36653793 10.1186/s12958-023-01054-0 PMC9847125

[14] Esteves SC , Santi D , Simoni M . An update on clinical and surgical interventions to reduce sperm DNA fragmentation in infertile men. Andrology. 2020;8(1):53–81. 31692293 10.1111/andr.12724

[15] Minhas S , Bettocchi C , Boeri L , Capogrosso P , Carvalho J , Cilesiz NC , et al. European Association of Urology guidelines on male sexual and reproductive health: 2021 update on male infertility. Eur Urol. 2021;80(5):603–620. 34511305 10.1016/j.eururo.2021.08.014

[16] ESHRE Guideline Group on RPL , Bender Atik R , Christiansen OB , Elson J , Kolte AM , Lewis S , et al. ESHRE guideline: recurrent pregnancy loss. Hum Reprod Open. 2018;2018(2):hoy004. 31486805 10.1093/hropen/hoy004 PMC6276652

[17] Schlegel PN , Sigman M , Collura B , De Jonge CJ , Eisenberg ML , Lamb DJ , et al. Diagnosis and treatment of infertility in men: AUA/ASRM guideline part I. J Urol. 2021;205(1):36–43. 33295257 10.1097/JU.0000000000001521

[18] Agarwal A , Majzoub A , Baskaran S , Panner Selvam MK , Cho CL , Henkel R , et al. Sperm DNA fragmentation: a new guideline for clinicians. World J Mens Health. 2020;38(4):412–471. 32777871 10.5534/wjmh.200128 PMC7502318

[19] Gao Y , Mutter‐Rottmayer E , Zlatanou A , Vaziri C , Yang Y . Mechanisms of post‐replication DNA repair. Genes. 2017;8(2):64. 10.3390/genes8020064 28208741 PMC5333053

[20] Ciccia A , Elledge SJ . The DNA damage response: making it safe to play with knives. Mol Cell. 2010;40(2):179–204. 20965415 10.1016/j.molcel.2010.09.019 PMC2988877

[21] Hanzlikova H , Gittens W , Krejcikova K , Zeng Z , Caldecott KW . Overlapping roles for PARP1 and PARP2 in the recruitment of endogenous XRCC1 and PNKP into oxidized chromatin. Nucleic Acids Res. 2017;45(5):2546–2557. 27965414 10.1093/nar/gkw1246 PMC5389470

[22] Pastink A , Lohman PH . Repair and consequences of double‐strand breaks in DNA. Mutat Res. 1999;428(1–2):141–156. 10517988 10.1016/s1383-5742(99)00042-3

[23] Khanna KK , Jackson SP . DNA double‐strand breaks: signaling, repair and the cancer connection. Nat Genet. 2001;27(3):247–254. 11242102 10.1038/85798

[24] Karagiannis TC , El‐Osta A . Double‐strand breaks: signaling pathways and repair mechanisms. Cell Mol Life Sci. 2004;61(17):2137–2147. 15338043 10.1007/s00018-004-4174-0 PMC11138614

[25] Mah L‐J , Vasireddy RS , Tang MM , Georgiadis GT , El‐Osta A , Karagiannis TC . Quantification of gammaH2AX foci in response to ionising radiation. J Vis Exp. 2010;(38):1957. 10.3791/1957 20372103 PMC3164074

[26] Valdiglesias V , Giunta S , Fenech M , Neri M , Bonassi S . γH2AX as a marker of DNA double strand breaks and genomic instability in human population studies. Mutat Res. 2013;753(1):24–40. 23416207 10.1016/j.mrrev.2013.02.001

[27] Agarwal A , Barbăroșie C , Ambar R , Finelli R . The impact of single‐ and double‐Strand DNA breaks in human spermatozoa on assisted reproduction. Int J Mol Sci. 2020 May 29;21(11):3882. 10.3390/ijms21113882 32485940 PMC7312948

[28] Ward WS , Coffey DS . DNA packaging and organization in mammalian spermatozoa: comparison with somatic cells. Biol Reprod. 1991;44(4):569–574. 2043729 10.1095/biolreprod44.4.569

[29] Balhorn R . A model for the structure of chromatin in mammalian sperm. J Cell Biol. 1982;93(2):298–305. 7096440 10.1083/jcb.93.2.298 PMC2112839

[30] Balhorn R . The protamine family of sperm nuclear proteins. Genome Biol. 2007;8(9):227. 17903313 10.1186/gb-2007-8-9-227 PMC2375014

[31] Miller D , Brinkworth M , Iles D . Paternal DNA packaging in spermatozoa: more than the sum of its parts? DNA, histones, protamines and epigenetics. Reproduction. 2010;139(2):287–301. 19759174 10.1530/REP-09-0281

[32] Gawecka JE , Ribas‐Maynou J , Benet J , Ward WS . A model for the control of DNA integrity by the sperm nuclear matrix. Asian J Androl. 2015;17(4):610–615. 25926613 10.4103/1008-682X.153853 PMC4492052

[33] Marcon L , Boissonneault G . Transient DNA strand breaks during mouse and human spermiogenesis new insights in stage specificity and link to chromatin remodeling. Biol Reprod. 2004;70(4):910–918. 14645105 10.1095/biolreprod.103.022541

[34] Aoki VW , Moskovtsev SI , Willis J , Liu L , Mullen JBM , Carrell DT . DNA integrity is compromised in protamine‐deficient human sperm. J Androl. 2005;26(6):741–748. 16291969 10.2164/jandrol.05063

[35] Ramos L , Daina G , Del Rey J , Ribas‐Maynou J , Fernández‐Encinas A , Martinez‐Passarell O , et al. Comprehensive preimplantation genetic screening and sperm deoxyribonucleic acid fragmentation from three males carrying balanced chromosome rearrangements. Fertil Steril. 2015;104(3):681–687. 26086421 10.1016/j.fertnstert.2015.05.033

[36] Hoeijmakers JHJ . DNA damage, aging, and cancer. N Engl J Med. 2009;361(15):1475–1485. 19812404 10.1056/NEJMra0804615

[37] Bui AD , Sharma R , Henkel R , Agarwal A . Reactive oxygen species impact on sperm DNA and its role in male infertility. Andrologia. 2018;50(8):e13012. 29644708 10.1111/and.13012

[38] Iwasaki A , Gagnon C . Formation of reactive oxygen species in spermatozoa of infertile patients. Fertil Steril. 1992;57(2):409–416. 1735495 10.1016/s0015-0282(16)54855-9

[39] Agarwal A , Sharma RK , Sharma R , Assidi M , Abuzenadah AM , Alshahrani S , et al. Characterizing semen parameters and their association with reactive oxygen species in infertile men. Reprod Biol Endocrinol. 2014;12:33. 24885775 10.1186/1477-7827-12-33 PMC4047553

[40] Farkouh A , Salvio G , Kuroda S , Saleh R , Vogiatzi P , Agarwal A . Sperm DNA integrity and male infertility: a narrative review and guide for the reproductive physicians. Transl Androl Urol. 2022;11(7):1023–1044. 35958895 10.21037/tau-22-149 PMC9360512

[41] Gunes S , Al‐Sadaan M , Agarwal A . Spermatogenesis, DNA damage and DNA repair mechanisms in male infertility. Reprod Biomed Online. 2015;31(3):309–319. 26206278 10.1016/j.rbmo.2015.06.010

[42] Sun S , Osterman MD , Li M . Tissue specificity of DNA damage response and tumorigenesis. Cancer Biol Med. 2019;16(3):396–414. 31565474 10.20892/j.issn.2095-3941.2019.0097 PMC6743622

[43] Iyama T , Wilson DM III . DNA repair mechanisms in dividing and non‐dividing cells. DNA Repair. 2013;12(8):620–636. 23684800 10.1016/j.dnarep.2013.04.015 PMC3720834

[44] Musson R , Gąsior Ł , Bisogno S , Ptak GE . DNA damage in preimplantation embryos and gametes: specification, clinical relevance and repair strategies. Hum Reprod Update. 2022;28(3):376–399. 35021196 10.1093/humupd/dmab046 PMC9071077

[45] Newman H , Catt S , Vining B , Vollenhoven B , Horta F . DNA repair and response to sperm DNA damage in oocytes and embryos, and the potential consequences in ART: a systematic review. Mol Hum Reprod. 2022;28(1):gaab071. 10.1093/molehr/gaab071 34954800

[46] Smith TB , Dun MD , Smith ND , Curry BJ , Connaughton HS , Aitken RJ . The presence of a truncated base excision repair pathway in human spermatozoa that is mediated by OGG1. J Cell Sci. 2013;126(Pt 6):1488–1497. 23378024 10.1242/jcs.121657

[47] Ahmadi A , Ng SC . Fertilizing ability of DNA‐damaged spermatozoa. J Exp Zool. 1999;284(6):696–704. 10531556 10.1002/(sici)1097-010x(19991101)284:6<696::aid-jez11>3.0.co;2-e

[48] González‐Marín C , Gosálvez J , Roy R . Types, causes, detection and repair of DNA fragmentation in animal and human sperm cells. Int J Mol Sci. 2012;13(11):14026–14052. 23203048 10.3390/ijms131114026 PMC3509564

[49] Jaroudi S , Kakourou G , Cawood S , Doshi A , Ranieri DM , Serhal P , et al. Expression profiling of DNA repair genes in human oocytes and blastocysts using microarrays. Hum Reprod. 2009;24(10):2649–2655. 19542543 10.1093/humrep/dep224

[50] Schlegel PN , Sigman M , Collura B , Dejonge CJ , Eisenberg ML , Lamb DJ , et al. Diagnosis and Treatment of Infertility in Men: AUA/ASRM Guideline [Internet]. 2020. https://www.auanet.org/guidelines‐and‐quality/guidelines/male‐infertility

[51] Ribas‐Maynou J , García‐Peiró A , Fernández‐Encinas A , Abad C , Amengual MJ , Prada E , et al. Comprehensive analysis of sperm DNA fragmentation by five different assays: TUNEL assay, SCSA, SCD test and alkaline and neutral comet assay. Andrology. 2013;1(5):715–722. 23843251 10.1111/j.2047-2927.2013.00111.x

[52] Sharma R , Iovine C , Agarwal A , Henkel R . TUNEL assay‐standardized method for testing sperm DNA fragmentation. Andrologia. 2021;53(2):e13738. 32706440 10.1111/and.13738

[53] Enciso M , Sarasa J , Agarwal A , Fernández JL , Gosálvez J . A two‐tailed comet assay for assessing DNA damage in spermatozoa. Reprod Biomed Online. 2009;18(5):609–616. 19549437 10.1016/s1472-6483(10)60003-x

[54] Rios JS , Coward RM , Hansen KR , Barnhart KT , Cedars MI , Legro RS , et al. Sperm deoxyribonucleic acid fragmentation: predictors, fertility outcomes, and assays among infertile males. Field Staff Rep. 2021;2(3):282–288. 10.1016/j.xfre.2021.06.003 PMC8441563 34553152

[55] Simon L , Brunborg G , Stevenson M , Lutton D , McManus J , Lewis SEM . Clinical significance of sperm DNA damage in assisted reproduction outcome. Hum Reprod. 2010;25(7):1594–1608. 20447937 10.1093/humrep/deq103

[56] Simon L , Lutton D , McManus J , Lewis SEM . Sperm DNA damage measured by the alkaline comet assay as an independent predictor of male infertility and in vitro fertilization success. Fertil Steril. 2011;95(2):652–657. 20864101 10.1016/j.fertnstert.2010.08.019

[57] Ribas‐Maynou J , García‐Peiró A , Abad C , Amengual MJ , Navarro J , Benet J . Alkaline and neutral comet assay profiles of sperm DNA damage in clinical groups. Hum Reprod. 2012;27(3):652–658. 22252081 10.1093/humrep/der461

[58] Garolla A , Cosci I , Bertoldo A , Sartini B , Boudjema E , Foresta C . DNA double strand breaks in human spermatozoa can be predictive for assisted reproductive outcome. Reprod Biomed Online. 2015;31(1):100–107. 25985994 10.1016/j.rbmo.2015.03.009

[59] Practice Committee of the American Society for Reproductive Medicine . The clinical utility of sperm DNA integrity testing: a guideline. Fertil Steril. 2013;99(3):673–677. 23391408 10.1016/j.fertnstert.2012.12.049

[60] Practice Committee of the American Society for Reproductive Medicine . Diagnostic evaluation of the infertile male: a committee opinion. Fertil Steril. 2015;103(3):e18–e25. 25597249 10.1016/j.fertnstert.2014.12.103

[61] Majzoub A , Agarwal A , Cho C‐L , Esteves SC . Sperm DNA fragmentation testing: a cross sectional survey on current practices of fertility specialists. Transl Androl Urol. 2017;6(Suppl 4):S710–S719. 29082205 10.21037/tau.2017.06.21 PMC5643631

[62] Santi D , Spaggiari G , Simoni M . Sperm DNA fragmentation index as a promising predictive tool for male infertility diagnosis and treatment management—meta‐analyses. Reprod Biomed Online. 2018;37(3):315–326. 30314886 10.1016/j.rbmo.2018.06.023

[63] Rey RA . Commentary on sperm DNA fragmentation testing clinical guideline. Transl Androl Urol. 2017;6(Suppl 4):S522–S524. 29082172 10.21037/tau.2017.03.19 PMC5643717

[64] Rex AS , Aagaard J , Fedder J . DNA fragmentation in spermatozoa: a historical review. Andrology. 2017;5(4):622–630. 28718529 10.1111/andr.12381 PMC5601286

[65] Setti AS , Braga DP , Provenza RR , Iaconelli A Jr , Borges E Jr . Oocyte ability to repair sperm DNA fragmentation: the impact of maternal age on intracytoplasmic sperm injection outcomes. Fertil Steril. 2021;116(1):123–129. 33589137 10.1016/j.fertnstert.2020.10.045

[66] Tesarik J , Greco E , Mendoza C . Late, but not early, paternal effect on human embryo development is related to sperm DNA fragmentation. Hum Reprod. 2004;19(3):611–615. 14998960 10.1093/humrep/deh127

[67] Kuroda S , Takeshima T , Takeshima K , Usui K , Yasuda K , Sanjo H , et al. Early and late paternal effects of reactive oxygen species in semen on embryo development after intracytoplasmic sperm injection. Syst Biol Reprod Med. 2020;66(2):122–128. 32063036 10.1080/19396368.2020.1720865

[68] Simon L , Murphy K , Shamsi MB , Liu L , Emery B , Aston KI , et al. Paternal influence of sperm DNA integrity on early embryonic development. Hum Reprod. 2014;29(11):2402–2412. 25205757 10.1093/humrep/deu228

[69] Zini A . Are sperm chromatin and DNA defects relevant in the clinic? Syst Biol Reprod Med. 2011;57(1–2):78–85. 21208147 10.3109/19396368.2010.515704

[70] Li Z , Wang L , Cai J , Huang H . Correlation of sperm DNA damage with IVF and ICSI outcomes: a systematic review and meta‐analysis. J Assist Reprod Genet. 2006;23(9–10):367–376. 17019633 10.1007/s10815-006-9066-9 PMC3455102

[71] Sugihara A , Van Avermaete F , Roelant E , Punjabi U , De Neubourg D . The role of sperm DNA fragmentation testing in predicting intra‐uterine insemination outcome: a systematic review and meta‐analysis. Eur J Obstet Gynecol Reprod Biol. 2020;244:8–15. 31707171 10.1016/j.ejogrb.2019.10.005

[72] Deng C , Li T , Xie Y , Guo Y , Yang Q‐Y , Liang X , et al. Sperm DNA fragmentation index influences assisted reproductive technology outcome: a systematic review and meta‐analysis combined with a retrospective cohort study. Andrologia. 2019;51(6):e13263. 30838696 10.1111/and.13263

[73] Simon L , Zini A , Dyachenko A , Ciampi A , Carrell DT . A systematic review and meta‐analysis to determine the effect of sperm DNA damage on in vitro fertilization and intracytoplasmic sperm injection outcome. Asian J Androl. 2017;19(1):80–90. 27345006 10.4103/1008-682X.182822 PMC5227680

[74] Zini A , Jamal W , Cowan L , Al‐Hathal N . Is sperm DNA damage associated with IVF embryo quality? A systematic review. J Assist Reprod Genet. 2011;28(5):391–397. 21327499 10.1007/s10815-011-9544-6 PMC3151360

[75] Zhao J , Zhang Q , Wang Y , Li Y . Whether sperm deoxyribonucleic acid fragmentation has an effect on pregnancy and miscarriage after in vitro fertilization/intracytoplasmic sperm injection: a systematic review and meta‐analysis. Fertil Steril. 2014;102(4):998–1005.e8. 25190048 10.1016/j.fertnstert.2014.06.033

[76] Ribas‐Maynou J , Yeste M , Becerra‐Tomás N , Aston KI , James ER , Salas‐Huetos A . Clinical implications of sperm DNA damage in IVF and ICSI: updated systematic review and meta‐analysis. Biol Rev Camb Philos Soc. 2021;96(4):1284–1300. 33644978 10.1111/brv.12700

[77] Esteves SC , Zini A , Coward RM , Evenson DP , Gosálvez J , Lewis SEM , et al. Sperm DNA fragmentation testing: summary evidence and clinical practice recommendations. Andrologia. 2021;53(2):e13874. 33108829 10.1111/and.13874 PMC7988559

[78] Osman A , Alsomait H , Seshadri S , El‐Toukhy T , Khalaf Y . The effect of sperm DNA fragmentation on live birth rate after IVF or ICSI: a systematic review and meta‐analysis. Reprod Biomed Online. 2015;30(2):120–127. 25530036 10.1016/j.rbmo.2014.10.018

[79] Evenson DP , Jost LK , Marshall D , Zinaman MJ , Clegg E , Purvis K , et al. Utility of the sperm chromatin structure assay as a diagnostic and prognostic tool in the human fertility clinic. Hum Reprod. 1999;14(4):1039–1049. 10221239 10.1093/humrep/14.4.1039

[80] Spanò M , Bonde JP , Hjøllund HI , Kolstad HA , Cordelli E , Leter G . Sperm chromatin damage impairs human fertility. The Danish first pregnancy planner study team. Fertil Steril. 2000 Jan;73(1):43–50. 10632410 10.1016/s0015-0282(99)00462-8

[81] Giwercman A , Lindstedt L , Larsson M , Bungum M , Spano M , Levine RJ , et al. Sperm chromatin structure assay as an independent predictor of fertility in vivo: a case‐control study. Int J Androl. 2010;33(1):e221–e227. 19840147 10.1111/j.1365-2605.2009.00995.x

[82] Chen Q , Zhao J‐Y , Xue X , Zhu G‐X . The association between sperm DNA fragmentation and reproductive outcomes following intrauterine insemination, a meta analysis. Reprod Toxicol. 2019;86:50–55. 30905832 10.1016/j.reprotox.2019.03.004

[83] Bungum M , Humaidan P , Axmon A , Spano M , Bungum L , Erenpreiss J , et al. Sperm DNA integrity assessment in prediction of assisted reproduction technology outcome. Hum Reprod. 2007;22(1):174–179. 16921163 10.1093/humrep/del326

[84] Chen Y , Li W , Chen X . The Association of Sperm DNA fragment and assisted reproductive outcomes: a meta‐analysis. Comput Math Methods Med. 2022;2022:1126616. 36158125 10.1155/2022/1126616 PMC9492328

[85] Simon L , Emery BR , Carrell DT . Review: diagnosis and impact of sperm DNA alterations in assisted reproduction. Best Pract Res Clin Obstet Gynaecol. 2017;44:38–56. 28935366 10.1016/j.bpobgyn.2017.07.003

[86] Lewis SEM . The place of sperm DNA fragmentation testing in current day fertility management. Middle East Fertil Soc J. 2013;18(2):78–82.

[87] ESHRE Guideline Group on RPL , Bender Atik R , Christiansen OB , Elson J , Kolte AM , Lewis S , et al. ESHRE guideline: recurrent pregnancy loss: an update in 2022. Hum Reprod Open. 2023;2023(1):hoad002. 36873081 10.1093/hropen/hoad002 PMC9982362

[88] Middelkamp S , van Tol HTA , Spierings DCJ , Boymans S , Guryev V , Roelen BAJ , et al. Sperm DNA damage causes genomic instability in early embryonic development. Sci Adv. 2020;6(16):eaaz7602. 32494621 10.1126/sciadv.aaz7602 PMC7159919

[89] Broer SL , Mol B , Dólleman M , Fauser BC , Broekmans FJM . The role of anti‐Müllerian hormone assessment in assisted reproductive technology outcome. Curr Opin Obstet Gynecol. 2010;22(3):193–201. 20407372 10.1097/GCO.0b013e3283384911

[90] Robinson L , Gallos ID , Conner SJ , Rajkhowa M , Miller D , Lewis S , et al. The effect of sperm DNA fragmentation on miscarriage rates: a systematic review and meta‐analysis. Hum Reprod. 2012;27(10):2908–2917. 22791753 10.1093/humrep/des261

[91] McQueen DB , Zhang J , Robins JC . Sperm DNA fragmentation and recurrent pregnancy loss: a systematic review and meta‐analysis. Fertil Steril. 2019;112(1):54–60.e3. 31056315 10.1016/j.fertnstert.2019.03.003

[92] Sun T‐C , Zhang Y , Li H‐T , Liu X‐M , Yi D‐X , Tian L , et al. Sperm DNA fragmentation index, as measured by sperm chromatin dispersion, might not predict assisted reproductive outcome. Taiwan J Obstet Gynecol. 2018;57(4):493–498. 30122567 10.1016/j.tjog.2018.06.003

[93] Green KA , Patounakis G , Dougherty MP , Werner MD , Scott RT Jr , Franasiak JM . Sperm DNA fragmentation on the day of fertilization is not associated with embryologic or clinical outcomes after IVF/ICSI. J Assist Reprod Genet. 2020;37(1):71–76. 31755002 10.1007/s10815-019-01632-5 PMC7000566

[94] Loloi J , Petrella F , Kresch E , Ibrahim E , Zini A , Ramasamy R . The effect of sperm DNA fragmentation on male fertility and strategies for improvement: a narrative review. Urology. 2022;168:3–9. 35705123 10.1016/j.urology.2022.05.036

[95] Goldhaber‐Fiebert JD , Brandeau ML . Evaluating cost‐effectiveness of interventions that affect fertility and childbearing: how health effects are measured matters. Med Decis Making. 2015;35(7):818–846. 25926281 10.1177/0272989X15583845 PMC4418217

[96] Humaidan P , Haahr T , Povlsen BB , Kofod L , Laursen RJ , Alsbjerg B , et al. The combined effect of lifestyle intervention and antioxidant therapy on sperm DNA fragmentation and seminal oxidative stress in IVF patients: a pilot study. Int Braz J Urol. 2022;48:131–156. 34472769 10.1590/S1677-5538.IBJU.2021.0604 PMC8691235

[97] Anifandis G , Bounartzi T , Messini CI , Dafopoulos K , Sotiriou S , Messinis IE . The impact of cigarette smoking and alcohol consumption on sperm parameters and sperm DNA fragmentation (SDF) measured by Halosperm(®). Arch Gynecol Obstet. 2014;290(4):777–782. 24840110 10.1007/s00404-014-3281-x

[98] Ranganathan P , Rao KA , Thalaivarasai BS . Deterioration of semen quality and sperm‐DNA integrity as influenced by cigarette smoking in fertile and infertile human male smokers‐a prospective study. J Cell Biochem. 2019;120(7):11784–11793. 30779221 10.1002/jcb.28458

[99] Axelsson J , Lindh CH , Giwercman A . Exposure to polycyclic aromatic hydrocarbons and nicotine, and associations with sperm DNA fragmentation. Andrology. 2022;10(4):740–748. 35234353 10.1111/andr.13170 PMC9310791

[100] Amor H , Jankowski PM , Dahadhah FW , Al Zoubi MS , Hammadeh ME . Impact of tobacco smoking in association with H2BFWT, PRM1 and PRM2 genes variants on male infertility. Andrologia. 2022;54(11):e14611. 36217675 10.1111/and.14611

[101] Esteves SC . Interventions to prevent sperm DNA damage effects on reproduction. Adv Exp Med Biol. 2019;1166:119–148. 31301050 10.1007/978-3-030-21664-1_8

[102] Jensen CFS , Østergren P , Dupree JM , Ohl DA , Sønksen J , Fode M . Varicocele and male infertility. Nat Rev Urol. 2017;14(9):523–533. 28675168 10.1038/nrurol.2017.98

[103] Schoor RA , Elhanbly SM , Niederberger C . The pathophysiology of varicocele‐associated male infertility. Curr Urol Rep. 2001;2(6):432–436. 12084227 10.1007/s11934-001-0035-7

[104] Zini A , Dohle G . Are varicoceles associated with increased deoxyribonucleic acid fragmentation? Fertil Steril. 2011;96(6):1283–1287. 22035729 10.1016/j.fertnstert.2011.10.016

[105] Abdelbaki SA , Sabry JH , Al‐Adl AM , Sabry HH . The impact of coexisting sperm DNA fragmentation and seminal oxidative stress on the outcome of varicocelectomy in infertile patients: a prospective controlled study. Arab J Urol. 2017;15(2):131–139. 29071142 10.1016/j.aju.2017.03.002 PMC5653613

[106] Lira Neto FT , Roque M , Esteves SC . Effect of varicocelectomy on sperm deoxyribonucleic acid fragmentation rates in infertile men with clinical varicocele: a systematic review and meta‐analysis. Fertil Steril. 2021;116(3):696–712. 33985792 10.1016/j.fertnstert.2021.04.003

[107] Esteves SC , Roque M , Agarwal A . Outcome of assisted reproductive technology in men with treated and untreated varicocele: systematic review and meta‐analysis. Asian J Androl. 2016;18(2):254–258. 26510504 10.4103/1008-682X.163269 PMC4770495

[108] Giagulli VA , Carbone MD . Varicocele correction for infertility: which patients to treat? Int J Androl. 2011;34(3):236–241. 20579135 10.1111/j.1365-2605.2010.01081.x

[109] Shah R , Agarwal A , Kavoussi P , Rambhatla A , Saleh R , Cannarella R , et al. Consensus and diversity in the management of varicocele for male infertility: results of a global practice survey and comparison with guidelines and recommendations. World J Mens Health. 2023;41(1):164–197. 35791302 10.5534/wjmh.220048 PMC9826919

[110] Negri L , Benaglia R , Monti E , Morenghi E , Pizzocaro A , Levi Setti PE . Effect of superoxide dismutase supplementation on sperm DNA fragmentation. Arch Ital Urol Androl. 2017;89(3):212–218. 28969406 10.4081/aiua.2017.3.212

[111] Boeri L , Lucignani G , Jannello LMI , Turetti M , Fulgheri I , Silvani C , et al. Clinically meaningful improvements in sperm DNA fragmentation severity in infertile men treated with superoxide dismutase supplementation: a single‐center experience. J Clin Med Res. 2022;11(21):6540. 10.3390/jcm11216540 PMC9656306 36362768

[112] Bahmyari R , Zare M , Sharma R , Agarwal A , Halvaei I . The efficacy of antioxidants in sperm parameters and production of reactive oxygen species levels during the freeze‐thaw process: a systematic review and meta‐analysis. Andrologia. 2020;52(3):e13514. 31967363 10.1111/and.13514

[113] Stenqvist A , Oleszczuk K , Leijonhufvud I , Giwercman A . Impact of antioxidant treatment on DNA fragmentation index: a double‐blind placebo‐controlled randomized trial. Andrology. 2018;6(6):811–816. 30298673 10.1111/andr.12547

[114] de Ligny W , Smits RM , Mackenzie‐Proctor R , Jordan V , Fleischer K , de Bruin JP , et al. Antioxidants for male subfertility. Cochrane Database Syst Rev. 2022;5(5):CD007411. 35506389 10.1002/14651858.CD007411.pub5 PMC9066298

[115] Knudtson JF , Sun F , Coward RM , Hansen KR , Barnhart KT , Smith J , et al. The relationship of plasma antioxidant levels to semen parameters: the males, antioxidants, and infertility (MOXI) randomized clinical trial. J Assist Reprod Genet. 2021;38(11):3005–3013. 34455507 10.1007/s10815-021-02301-2 PMC8609082

[116] Kuchakulla M , Ramasamy R . Re: the effect of antioxidants on male factor infertility: the males, antioxidants, and infertility (MOXI) randomized clinical trial. Eur Urol. 2021;79(1):159–160. 32847700 10.1016/j.eururo.2020.08.008

[117] Kooshesh L , Bahmanpour S , Zeighami S , Nasr‐Esfahani MH . Effect of Letrozole on sperm parameters, chromatin status and ROS level in idiopathic oligo/Astheno/Teratozoospermia. Reprod Biol Endocrinol. 2020;18(1):47. 32404173 10.1186/s12958-020-00591-2 PMC7218838

[118] Lispi M , Drakopoulos P , Spaggiari G , Caprio F , Colacurci N , Simoni M , et al. Testosterone serum levels are related to sperm DNA fragmentation index reduction after FSH Administration in Males with idiopathic infertility. Biomedicines. 2022;10(10):2599. 10.3390/biomedicines10102599 36289860 PMC9599665

[119] La Vignera S , Condorelli RA , Duca Y , Mongioi LM , Cannarella R , Giacone F , et al. FSH therapy for idiopathic male infertility: four schemes are better than one. Aging Male. 2020;23(5):750–755. 30942139 10.1080/13685538.2019.1590696

[120] Akang EN , Dosumu OO , Ogbenna AA , Akpan U‐OU , Ezeukwu JC , Odofin MO , et al. The impact of dolutegravir‐based combination antiretroviral therapy on the spermatozoa and fertility parameters of men living with human immunodeficiency virus. Andrologia. 2022;54(11):e14621. 36261884 10.1111/and.14621 PMC9722517

[121] Boeri L , Capogrosso P , Ventimiglia E , Pederzoli F , Cazzaniga W , Chierigo F , et al. High‐risk human papillomavirus in semen is associated with poor sperm progressive motility and a high sperm DNA fragmentation index in infertile men. Hum Reprod. 2019;34(2):209–217. 30517657 10.1093/humrep/dey348

[122] Ambar RF , Agarwal A , Majzoub A , Vij S , Tadros NN , Cho CL , et al. The use of testicular sperm for intracytoplasmic sperm injection in patients with high sperm DNA damage: a systematic review. World J Mens Health. 2021;39(3):391–398. 32648379 10.5534/wjmh.200084 PMC8255394

[123] Esteves SC , Coimbra I , Hallak J . Surgically retrieved spermatozoa for ICSI cycles in non‐azoospermic males with high sperm DNA fragmentation in semen. Andrology. 2023;11(8):1613–1634. 10.1111/andr.13405 36734283

[124] Zhang J , Xue H , Qiu F , Zhong J , Su J . Testicular spermatozoon is superior to ejaculated spermatozoon for intracytoplasmic sperm injection to achieve pregnancy in infertile males with high sperm DNA damage. Andrologia. 2019;51(2):e13175. 30474187 10.1111/and.13175

[125] Tharakan T , Bettocchi C , Carvalho J , Corona G , Jones TH , Kadioglu A , et al. European Association of Urology guidelines panel on male sexual and reproductive health: a clinical consultation guide on the indications for performing sperm DNA fragmentation testing in men with infertility and testicular sperm extraction in nonazoospermic men. Eur Urol Focus. 2022;8(1):339–350. 33422457 10.1016/j.euf.2020.12.017

[126] Farkouh A , Agarwal A , Hamoda TA‐AA‐M , Kavoussi P , Saleh R , Zini A , et al. Controversy and consensus on the Management of Elevated Sperm DNA fragmentation in male infertility: a global survey, current guidelines, and expert recommendations. World J Mens Health. 2023;41:809–847. 10.5534/wjmh.230008 37118965 PMC10523126

[127] Alipour H , Van Der Horst G , Christiansen OB , Dardmeh F , Jørgensen N , Nielsen HI , et al. Improved sperm kinematics in semen samples collected after 2 h versus 4‐7 days of ejaculation abstinence. Hum Reprod. 2017;32(7):1364–1372. 28531319 10.1093/humrep/dex101

[128] Agarwal A , Gupta S , Du Plessis S , Sharma R , Esteves SC , Cirenza C , et al. Abstinence time and its impact on basic and advanced semen parameters. Urology. 2016;94:102–110. 27196032 10.1016/j.urology.2016.03.059

[129] Bahadur G , Almossawi O , Zeirideen Zaid R , Ilahibuccus A , Al‐Habib A , Muneer A , et al. Semen characteristics in consecutive ejaculates with short abstinence in subfertile males. Reprod Biomed Online. 2016;32(3):323–328. 26776821 10.1016/j.rbmo.2015.11.021

[130] Sokol P , Drakopoulos P , Polyzos NP . The effect of ejaculatory abstinence interval on sperm parameters and clinical outcome of ART. A systematic review of the literature. J Clin Med Res. 2021;10(15):3213. 10.3390/jcm10153213 PMC8347289 34361997

[131] Ayad BM , der Horst GV , Plessis SSD . Revisiting the relationship between the ejaculatory abstinence period and semen characteristics. Int J Fertil Steril. 2018;11(4):238–246. 29043697 10.22074/ijfs.2018.5192 PMC5641453

[132] Manna C , Barbagallo F , Manzo R , Rahman A , Francomano D , Calogero AE . Sperm parameters before and after swim‐up of a second ejaculate after a short period of abstinence. J Clin Med Res. 2020;9(4):1029. 10.3390/jcm9041029 PMC7231087 32260592

[133] Barbagallo F , Cannarella R , Crafa A , Manna C , La Vignera S , Condorelli RA , et al. The impact of a very short abstinence period on conventional sperm parameters and sperm DNA fragmentation: a systematic review and meta‐analysis. J Clin Med Res. 2022;11(24):7303. 10.3390/jcm11247303 PMC9782170 36555920

[134] Leandri RD , Gachet A , Pfeffer J , Celebi C , Rives N , Carre‐Pigeon F , et al. Is intracytoplasmic morphologically selected sperm injection (IMSI) beneficial in the first ART cycle? A multicentric randomized controlled trial. Andrology. 2013;1(5):692–697. 23788532 10.1111/j.2047-2927.2013.00104.x

[135] Hozyen M , Hasanen E , Elqusi K , ElTanbouly S , Gamal S , Hussin AG , et al. Reproductive outcomes of different sperm selection techniques for ICSI patients with abnormal sperm DNA fragmentation: a randomized controlled trial. Reprod Sci. 2022;29(1):220–228. 34076869 10.1007/s43032-021-00642-y

[136] González‐Ravina C , Santamaría‐López E , Pacheco A , Ramos J , Carranza F , Murria L , et al. Effect of sperm selection by magnetic‐activated cell sorting in D‐IUI: a randomized control trial. Cells. 2022;11(11):1794. 10.3390/cells11111794 35681488 PMC9180176

[137] Kuroda S , Karna KK , Kaiyal RS , Cannarella R , Lundy SD , Vij SC , et al. Novel sperm chromatin dispersion test with artificial intelligence‐aided halo evaluation: a comparison study with existing modalities. Andrology. 2023;11(8):1581–1592. 10.1111/andr.13436 37002661

[138] McCallum C , Riordon J , Wang Y , Kong T , You JB , Sanner S , et al. Deep learning‐based selection of human sperm with high DNA integrity. Commun Biol. 2019;2:250. 31286067 10.1038/s42003-019-0491-6 PMC6610103

